# Association of self-efficacy, risk attitudes, and time preferences with health-related quality of life and functioning after total hip or knee replacement – Results of the MobilE-TRA 2 cohort

**DOI:** 10.1186/s12955-025-02374-y

**Published:** 2025-04-23

**Authors:** Sebastian Fuchs, Lars Schwettmann, Benedict Katzenberger, Alexander Paulus, Boris Michael Holzapfel, Johanna Theresia Biebl, Martin Weigl

**Affiliations:** 1 Department of Orthopaedics and Trauma Surgery, LMU University Hospital, LMU Munich, Munich, Germany; 2 Institute for Medical Information Processing, Biometry and Epidemiology (IBE), Faculty of Medicine, LMU Munich, Munich, Germany; 3 Pettenkofer School of Public Health, Munich, Germany; 4 Department of Health Services Research, Faculty VI - School of Medicine and Health Sciences, Carl von Ossietzky Universität Oldenburg, Oldenburg, Germany; 5 Institute of Health Economics and Health Care Management (IGM), Helmholtz Zentrum München (GmbH) – German Research Center for Environmental Health, Neuherberg, Germany; 6 Orthopaedisches Fachzentrum Weilheim-Garmisch-Starnberg-Penzberg, Weilheim, Germany

**Keywords:** Self-efficacy, Risk attitudes, Time preferences, Arthroplasty, Health-related quality of life, Functioning

## Abstract

**Background:**

While total hip and knee replacement (THR/TKR) surgery are effective measures to restore functioning and reduce pain in patients with severe osteoarthritis (OA), long-term treatment effects vary among patients. Following behavioral economic theory, these differences may be partially attributed to the impact of personality traits on individual strategies to approach post-surgical challenges. This study explored the associations between self-efficacy, willingness to take risk regarding health (H-WTTR), and future orientation, and the 3-month course of health-related quality of life (HRQoL) and OA-specific health status.

**Methods:**

As part of the prospective and observational MobilE-TRA 2 cohort study, 147 patients aged 60 years and older were assessed by self-administered questionnaires before and three months after THR/TKR at a single German hospital. As indicators for the surgical outcome, HRQoL was assessed by the EuroQol Five-Dimensional Five-Level Questionnaire (EQ-5D-5L), including the visual analogue scale (EQ-VAS), and functioning was assessed by the Western Ontario and McMaster Universities Osteoarthritis Index (WOMAC) using the global score, function score, and pain score. All WOMAC scores were transformed into scales with 0 = worst health and 100 = best health. Self-efficacy was measured using the General Self-Efficacy Short Scale. H-WTTR and future orientation were assessed by single-item questions on 11-point Likert scales. The associations between these personality traits and the 3-month change in the outcome scores were analyzed using linear regression models for THR and TKR respectively.

**Results:**

In THR patients a one-point-increase in self-efficacy was associated with improvements in EQ-5D-5L (β=0.0704; *p*=0.0099), WOMAC global (β=6.6337; *p*=0.0139), WOMAC function (β=8.2557; *p*=0.0046), and WOMAC pain (β=5.9994; *p*=0.0232). For TKR, only the association of self-efficacy with the EQ-VAS change-score was significant (β=5.8252; *p*=0.0482). Self-efficacy demonstrated weak positive, but not significant associations with all WOMAC scores and a negative association close to zero with the EQ-Index. H-WTTR and future orientation showed no significant associations to changes of the outcome scores.

**Conclusions:**

Self-efficacy appears to be a prognostic factor for better THR/TKR outcomes after three months. If these findings can be confirmed in further research, strategies to improve self-efficacy should be considered in prehabilitation programs.

**Trial registration:**

Not applicable.

**Supplementary Information:**

The online version contains supplementary material available at 10.1186/s12955-025-02374-y.

## Background

Total hip and knee replacement (THR/TKR) combined are the most frequent surgeries in German hospitals [1]. The positive effects of THR/TKR for patients with severe osteoarthritis (OA) are well documented [2–4]. In all age groups, THR/TKR improve physical functioning and health-related quality of life (HRQoL) and reduce pain [5]. However, long-term treatment effects vary considerably among patients [6]. The reasons are still incompletely understood.

Previous literature highlights the importance of early rehabilitation in THR/TKR for the long-term improvement of HRQoL and functioning [7, 8]. Physical therapy and home exercises after surgery improve mobility and physical functioning and reduce pain [9, 10]. Better adherence to recommended exercise improves the long-term outcomes in patients with knee and/or hip OA [11]. Accordingly, personality traits associated with adherence to treatment recommendations may contribute to variations in HRQoL and functioning after THR/TKR.

Economic theory considers beneficial health behavior as an investment in good health [12]. However, individuals tend to deviate from favorable healthy behaviors despite ample knowledge about the consequences [13]. Behavioral economics integrates concepts from various other scientific fields including psychology, anthropology, and neuroscience to address and explain these deviations. This analysis focuses on self-efficacy, willingness to take risk in the context of health (H-WTTR), and time preferences, captured by future orientation, three often discussed personality traits in behavioral economics [13–17] and established determinants of health behavior [18–21]. Consequently, they might influence OA patients’ strategies to approach challenges after THR/TKR and thus affect HRQoL and functioning.

Prior research on personality traits as prognostic factors for THR/TKR outcomes produced mixed findings. Whereas higher preoperative self-efficacy predicted improvements in OA-specific health status three months after THR [22], another examination of THR/TKR patients six weeks after surgery could not find evidence for this association [23]. A pilot study on a self-efficacy intervention before THR/TKR delivered inconclusive results [24].

Direct evidence linking future orientation or H-WTTR with THR/TKR is still missing. However, studies conducted in other populations have identified future orientation as a determinant of a more physically active lifestyle and greater adherence to physical activity advice and dietary recommendations [25–27]. Given that physical activity and avoiding obesity are associated with favorable outcomes following THR/TKR, it is plausible that future orientation may likewise contribute to improved postoperative results in this patient group. Risk preferences were linked to unhealthy behaviors such as smoking, but also to health-promoting behaviors such as physical activity [21, 27].

The aim of this study was to examine preoperative self-efficacy, H-WTTR, and future orientation as determinants of changes in different domains of HRQoL and OA-specific health status from the time of surgery to the three-month follow-up in patients with hip or knee OA undergoing THR/TKR.

## Methods

### Study design

This analysis is part of the longitudinal observational cohort study MobilE-TRA 2, conducted at the LMU University Hospital Munich in Germany. MobilE-TRA 2 examines determinants of patient outcomes using insights from behavioral economics to inform care practices and improve mobility and participation in older adults. The study comprises two subprojects targeting distinct disease groups: OA – the focus of this publication – and vertigo, dizziness, and balance disorders. The OA subproject was carried out at the Musculoskeletal University Center Munich (MUM), a division of the LMU University Hospital Munich [28, 29].

This subproject involved OA patients aged 60 and older presenting for THR/TKR at the MUM. Exclusion criteria were insufficient command of the German language or other insufficient skills to participate and fill out the questionnaire, such as impaired visual or hearing function. Eligibility was checked by a member of the study team.

The sample size was calculated based on the EuroQol visual analogue scale (EQ-VAS) assuming a minimum important change (MIC) of 9 for THR patients, 8 for TKR, and a SD of 18 for both groups [30]. The MIC represents the minimal mean within-person change that is perceived by patients as important [31]. 38 THR and 47 TKR patients were necessary to estimate the mean with a power of 0.85 (α= 0.05). Two follow-ups and 20% loss to follow-up, derived from previous projects, required 132 (59 THR, 73 TKR) patients at baseline.

The study design was published beforehand [28] and approved by the ethics committee of the medical faculty at the Ludwig Maximilian University Munich. All participants provided written informed consent. This manuscript adheres to the STROBE Statement [32].

### Setting, participants, and data collection

Assessments before (baseline) and three months after THR/TKR (follow-up) were conducted between November 2020 and October 2022. The study was introduced during clinic visits, at the ward, or by telephone. No participation incentive was offered. During recruitment, all eligible and available patients were contacted at the MUM for a complete survey. Interested patients received baseline study materials either personally or by mail. The baseline questionnaire completion period was within the last 14 days before THR/TKR. All questionnaires were paper-based and designed for self-administration, allowing participants to complete them independently at home or the MUM if their hospital stay commenced one day before their scheduled THR/TKR.

Follow-ups were sent by postal mail. Two reminders, by mail and telephone, followed in 9-day intervals. Reasons for premature withdrawal were obtained during these telephone calls. Throughout all assessments, participants could contact the study team if they required clarification on the questionnaires. Upon return, all questionnaires were checked for completeness and plausibility. In cases of missing or unclear responses, patients were contacted again. These extensive quality control procedures and comprehensive participant support contributed to high response rates and optimal data quality.

### Intervention

THR/TKR were performed according to the German guidelines for THR/TKR indications of the Association of the Scientific Medical Societies in Germany and the hospital standard procedures [33, 34]. Physiotherapy started on the day of surgery or the following day. Further physiotherapeutic treatments were scheduled daily on weekdays and once per weekend.

Median hospital stay was 8 days for THR and TKR, respectively. Afterward, patients underwent approximately three weeks of multimodal inpatient rehabilitation, the typical postoperative care provided in Germany after THR/TKR [33, 34].

### Measures

#### Outcomes

HRQoL was measured using the EuroQol Five-Dimensional Five-Level Questionnaire (EQ-5D-5L), its dimensions range from 0 (best) to 5 (worst), and the EuroQol visual analogue scale (EQ-VAS) ranging from 0 (worst) to 100 (best) [35]. The EQ-5D-5L utility index (EQ-index) ranging from − 0.661 (worst) to 1 (best) was calculated with the German value set for population-based utilities [36]. To facilitate the interpretation of the results, the MIC was included as a reference value for each outcome. According to previous literature, the MIC for the EQ-index was reported as 0.106 for improvements after THR and 0.090 after TKR [37]. On the EQ-VAS, the MIC was defined as a 9-point change for THR and an 8-point change for TKR [30].

OA-specific health status was assessed using the Western Ontario and McMaster Universities Osteoarthritis Index (WOMAC) in Likert scale format [38, 39]. Based on 24 questions, separated into 3 different scales (pain, stiffness, physical function) the WOMAC was specifically developed to evaluate hip and knee OA [38]. The WOMAC global score was implemented by adding up all three equally weighted subscales [40]. Following methodological recommendations, WOMAC scores were transformed to a range from 0 (worst) to 100 (best) to facilitate interpretation and comparison between the scales [41]. As recommended in the literature, the MIC based on a change from their respective mean baseline values is 14% for the WOMAC global score, 13.9% for the functioning scale and 15.5% for the pain scale [42]. The respective values will be reported in the results section.

#### Personality traits

General self-efficacy was measured by the validated General Self-Efficacy Short Scale, ranging from 1 (very low) to 5 (very high) as the mean of three items on self-efficacy [43]. H-WTTR was assessed by a single validated question on an 11-point Likert Scale regarding willingness to take risk in the context of health from 0 (‘not at all ready to take risks’) to 10 (‘very likely to take risks’) [21]. Two single-question items captured time preferences. Future orientation was assessed by ‘I am ready to sacrifice my well-being in the present to achieve certain results in the future’ on an 11-point Likert scale [44], where higher values represent a stronger focus on the future. Present orientation was surveyed by ‘I am only concerned about the present, because I trust that things will work themselves out in the future’ from 1 (totally disagree) to 5 (totally agree) [44].

#### Covariables

Clinical data on body mass index (BMI), prior THR/TKR surgeries, and the American Society of Anesthesiologists (ASA) score [45] was collected from the clinical information system. The ASA score classifies preoperative physical health into five groups based on medical history, physical examination, and diagnostic tests: healthy, mild systemic disease, severe systemic disease, incapacitating disease constantly threatening life, and moribund (life expectancy < 24 hours). The ASA score is associated with perioperative risks.

Questionnaires assessed age, sex, education, marital status, and chronic comorbidities. Education was categorized by years of formal education: lower secondary (up to 9 years), intermediate secondary (10 - 11 years), upper secondary (12–13 years), and tertiary education (college/university degree) [29]. The prespecified list in Supplementary File 1 - Table S1 assessed the number of comorbidities. Free text on further diseases was classified according to the International Statistical Classification of Diseases and Related Health Problems 10th revision (ICD-10) [46]. The number of comorbidities was categorized into 0, 1, 2, 3, and 4 or more conditions.

**Fig. 1:**
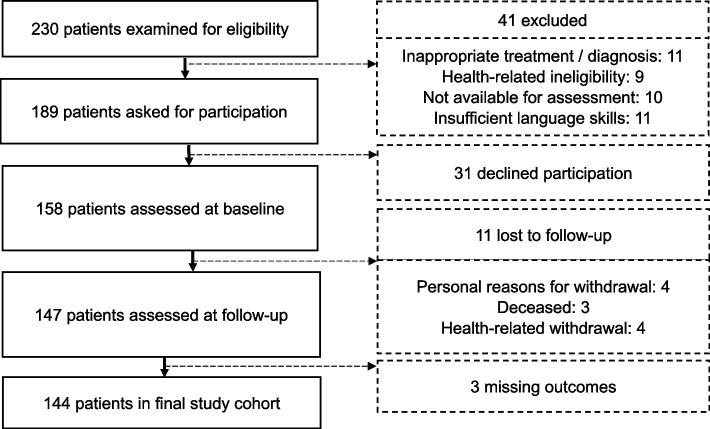
Patient Flow Diagram

Based on three directed acyclic graphs (DAGs), a confirmatory covariate selection identified the minimal sufficient adjustment sets for HRQoL and OA-specific functioning and pain. A separate literature review informed the model’s interrelations and effect directions. Hence, regressions control sufficiently for potential confounding while avoiding over-adjustment or collider bias [47]. Supplementary File 2 - Figures 1 - 3 provide all DAG models with references. The DAG identified sex, age, and number of diseases as essential co-variates in all regression models and additionally education in estimations of HRQoL.

### Statistical methods

Summary statistics were calculated separately for the total, THR, and TKR samples. Preoperative differences between THR and TKR patients were tested for significance using t-test, χ^2^-test, and Kruskal–Wallis test, within-group between the assessments using t-tests, and Wilcoxon signed-rank tests. Baseline associations between personality traits were calculated by the Pearson correlation coefficient.

To examine the association between the personality traits self-efficacy, H-WTTR, and future orientation at baseline and change scores of HRQoL and OA-specific health status from baseline to follow-up, linear regression models were computed for EQ-5D index, EQ-VAS, WOMAC global, WOMAC function, and WOMAC pain. Analyses were separated by THR/TKR and outcome. A sensitivity analysis replaced future orientation with present orientation to check the robustness of results regarding time preferences. The models were adjusted for the baseline values of the outcome measure [22, 23, 48].

Only complete cases were analyzed, as missing data only affected three values in three different patients. According to Jakobsen et al., multiple imputation was deemed unnecessary, due to the low impact of missing data [49]. Supplementary File 3 - Table S2 provides an overview of missing data for all key variables.

Sensitivity analysis addressed potential selection bias of healthier patients during the SARS-CoV- 2 pandemic due to limited availability of respirators and ICU beds. Univariate linear regression was used to analyze changes in the ASA score over time. The significance level was set at 5%.

Analyses were conducted in R version 4.2.2 [50]. DAGs were created and analyzed using DAGitty [51].

## Results

### Participants

During the recruitment period, 230 consecutive patients with THR/TKR were assessed for eligibility. 41 patients were ineligible and 31 declined participation. Of the remaining 158, 147 patients (93%) replied at follow-up. 11 patients were lost to follow-up. Missing outcome data required the exclusion of 3 more patients. Figure 1 gives detailed information on the patient flow.

### Patient characteristics

Table 1 shows baseline patient characteristics. The study population was composed of 75 patients with THR and 69 with TKR. THR/TKR cohorts did not differ significantly, except for the worse ASA score before THR.

**Table 1:** Preoperative patient characteristics

**Measure**	**Value**	**Overall**	**Hip**	**Knee**	***p*-value**
n		144	75	69	
Age median [IQR]		72 [67, 79]	72 [67, 78]	72 [66, 79]	0.898^c^
Female, n (%)		81 (56.2)	45 (60.0)	36 (52.2)	0.437^a^
Self-efficacy (mean (SD))		4.03 (0.87)	4.03 (0.83)	4.01 (0.93)	0.935^a^
Health-related willingness to take risk (mean (SD))		4.90 (2.40)	4.72 (2.36)	5.10 (2.43)	0.342^a^
Future orientation (mean (SD))		6.30 (2.46)	6.28 (2.50)	6.32 (2.44)	0.924^c^
Number of diseases, n (%)	0	25 (17.4)	13 (17.3)	12 (17.4)	0.135^b^
	1	38 (26.4)	15 (20.0)	23 (33.3)	
	2	31 (21.5)	15 (20.0)	16 (23.2)	
	3	19 (13.2)	10 (13.3)	9 (13.0)	
	> 4	32 (21.5)	22 (29.3)	9 (13.0)	
BMI median [IQR]		26.9 [23.2, 30.1]	26.4 [22.0, 29.7]	27.5 [24.6, 30.4]	0.059^c^
ASA score, n (%)	1	4 (2.8)	3 (4.0)	1 (1.4)	0.028^b^
	2	52 (36.1)	19 (25.3)	33 (47.8)	
	3	85 (59.0)	52 (69.3)	33 (47.8)	
	4	3 (2.1)	1 (1.3)	2 (2.9)	
Previous THR/TKR, n (%)	Yes	52 (36.1)	24 (32.0)	28 (40.6)	0.370^b^
Years of education, n (%)	≤ 9	60 (41.7)	31 (41.3)	29 (42.0)	0.296^b^
	10 - 11	35 (24.3)	14 (18.7)	21 (30.4)	
	12 - 13	10 (6.9)	6 (8.0)	4 (5.8)	
	> 13	39 (27.1)	24 (32.0)	15 (21.7)	

*IQR* Interquartile range, *SD* Standard deviation, general self-efficacy (1–5) higher scores indicate higher self-efficacy, health-related willingness to take risk (0–10) lower values represent more risk aversion, future orientation (0–10) higher values indicate a stronger orientation on the future, *BMI* body mass index, *ASA score (1–5)* American Society of Anesthesiologists score assessing the physical status of a patient before surgery with values representing (1) healthy, (2) mild systemic disease, (3) severe systemic disease, (4) life-threatening severe systemic disease, (5) moribund patient respectively, *THR/TKR: (0, 1)* Total hip or knee replacement, indicates if a patient already had a total hip or knee replacement surgery at baseline assessment

*P*-values calculated using: ^a^ t-test for unpaired samples, ^b^ Chi-squared test, ^c^ Kruskal–Wallis test

### Treatment outcome

Table 2 presents HRQoL and WOMAC scores at baseline and follow-up. Both THR and TKR patients improved significantly, with greater improvements in THR patients.

**Table 2:** Quality of life and WOMAC scores at baseline and after three months

		**THR**			**TKR**	
**Measure**	**Baseline**	**Follow-up**	***p*-value**	**Baseline**	**Follow-up**	***p*-value**
n	75	75		69	69	
EQ-5D-5L utility index (median [IQR])	0.48 [0.33, 0.77]	0.88 [0.76, 0.94]	< 0.001^b^	0.67 [0.46, 0.85]	0.88 [0.74, 0.94]	< 0.001^b^
EQ-VAS (mean (SD))	56.80 (21.95)	69.44 (21.65)	< 0.001^a^	60.64 (19.81)	71.04 (18.82)	0.002^a^
WOMAC global (mean (SD))	41.93 (17.94)	74.22 (18.45)	< 0.001^a^	54.36 (19.90)	70.46 (19.05)	< 0.001^a^
WOMAC functioning (mean (SD))	42.90 (18.52)	76.02 (19.99)	< 0.001^a^	58.55 (20.20)	74.10 (20.16)	< 0.001^a^
WOMAC pain (mean (SD))	44.40 (19.85)	80.82 (18.83)	< 0.001^a^	53.62 (21.28)	74.24 (18.02)	< 0.001^a^
WOMAC stiffness (mean (SD))	38.50 (24.64)	65.83 (23.01)	< 0.001^a^	50.91 (27.06)	63.04 (24.86)	0.007^a^

All WOMAC scores were rescaled to 0–100 and inverted where high values represent a better health status; WOMAC global: aggregated score of all three WOMAC subscales equally weighted

*THR* Total hip replacement, *TKR* Total knee replacement, *EQ-5D-5L* EuroQol Five-Dimensional Five-Level Questionnaire, *EQ-VAS* EuroQol visual analogue scale, *WOMAC* Western Ontario and McMaster Universities Osteoarthritis Index, *IQR* Interquartile range, *SD* Standard deviation

*P*-values for differences between baseline and follow-up within groups of THR and TKR were calculated using: ^a^ t-test for paired samples, ^b^ Wilcoxon signed-rank test

Based on the respective baseline values and recommended thresholds from the literature, the calculated MICs for THR were 5.87 for the WOMAC global, 5.96 for the WOMAC functioning, and 6.88 for the WOMAC pain. For TKR, the corresponding MICs were 7.61 for the WOMAC global, 8.14 for the WOMAC functioning, and 8.31 for the WOMAC pain [42].

### EQ-5D-5L dimensions

THR patients improved in all dimensions, particularly mobility and pain (Table 3). TKR also improved in mobility and pain, but the follow-up scores in self-care, usual activities, and anxiety/depression were similar to baseline.

**Table 3:** Comparison of EQ-5D-5L at baseline and after three months between THR and TKR

		**Baseline**	**Follow-up**	**Change**	**T2 vs. T1**	**Differences in change scores**
	**Group**	**Mean (SD)**	**Mean (SD)**		***p*-value**	***p*-value**
Mobility	THR	3.28 (0.97)	2.00 (1.08)	− 1.28	< 0.001^a^	0.053^b^
	TKR	3.03 (1.04)	2.14 (1.06)	− 0.88	< 0.001^a^	
Self-care	THR	1.67 (0.96)	1.35 (0.60)	− 0.32	0.016^a^	0.038^b^
	TKR	1.35 (0.76)	1.32 (0.65)	− 0.03	0.811^a^	
Usual activity	THR	2.56 (1.19)	1.89 (1.02)	− 0.67	< 0.001^a^	< 0.001^b^
	TKR	1.91 (1.07)	2.00 (1.03)	0.09	0.627^a^	
Pain	THR	3.67 (0.68)	2.13 (0.86)	− 1.53	< 0.001^a^	0.001^b^
	TKR	3.30 (0.85)	2.36 (0.92)	− 0.94	< 0.001^a^	
Anxiety/depression	THR	1.60 (0.85)	1.39 (0.68)	− 0.21	0.092^a^	0.363^b^
	TKR	1.51 (0.83)	1.42 (0.69)	− 0.09	0.507^a^	

Higher values represent greater restrictions of HRQoL, negative change-scores imply improvement of symptoms

*THR* Total hip replacement, *TKR* Total knee replacement, *EQ-5D-5L dimensions* EuroQol Five-Dimensional Five-Level Questionnaire dimensions (1 - 5)

*P*-values for differences between baseline and follow-up within groups of THR and TKR were calculated using ^a^ t-test for paired samples. *P*-values for differences in change scores were calculated using ^b^ t-test for unpaired samples

### Correlations of personality traits with HRQoL and WOMAC at baseline

Table 4 presents the baseline correlations of personality traits with the EQ and WOMAC scores.

**Table 4:** Correlations of personality traits with HRQoL and WOMAC scores at baseline

		**EQ-Index**	**EQ-VAS**	**WOMAC** **global**	**WOMAC** **function**	**WOMAC** **pain**
	Self-efficacy	0.435 ***	0.385 ***	0.232 *	0.306 **	0.237 *
**THR patients**	Health-related willingness to take risk	0.228 *	0.213	0.034	0.118	0.077
	Future orientation	0.207	0.358 **	0.188	0.325 **	0.153
	Self-efficacy	0.471 ***	0.344 **	0.412 ***	0.491 ***	0.332 **
**TKR patients**	Health-related willingness to take risk	0.296 *	0.352 **	0.065	0.067	0.052
	Future orientation	− 0.070	0.188	0.067	0.116	0.119

Pearson correlations between personality traits and all outcomes

*THR* Total hip replacement, *TKR* Total knee replacement, *EQ-5D-5L* EuroQol Five-Dimensional Five-Level Questionnaire, *EQ-VAS* EuroQol visual analogue scale, *WOMAC* Western Ontario and McMaster Universities Osteoarthritis Index, General self-efficacy (1–5) higher scores indicate higher self-efficacy, health-related willingness to take risk (0–10) lower values represent risk aversion, future orientation (0–10) higher values indicate a stronger orientation on the future

Significance: *p < 0.05, **p < 0.01, ***p < 0.001; in bold if the result was at least significant at *p* < 0.05 level

In THR patients, the correlations were consistently positive, with self-efficacy significantly associated with EQ-index (*r*= 0.435; *p*= 0.000), EQ-VAS (*r*= 0.385; *p*= 0.001), WOMAC global (*r*= 0.232; *p*= 0.045), WOMAC functioning (*r*= 0.306; *p*= 0.008) and WOMAC pain (*r*= 0.237; *p*= 0.041). For H-WTTR only EQ-index (*r*= 0.228; *p*= 0.0496) correlated significantly. Future orientation was significantly associated with EQ-VAS (*r*= 0.385; *p*= 0.002) and WOMAC functioning (*r*= 0.325; *p*= 0.005).

Regarding TKR, positive correlations also prevailed. Self-efficacy had significant associations with EQ-index (*r*= 0.471; *p*= 0.000), EQ-VAS (*r*= 0.344; *p*= 0.004), WOMAC global score (*r*= 0.412; *p*= 0.000), WOMAC functioning (*r*= 0.491; *p*= 0.000) and WOMAC pain (*r*= 0.332; *p*= 0.005). H-WTTR correlated significantly with EQ-index (*r*= 0.296; *p*= 0.014) and EQ-VAS (*r*= 0.352; *p*= 0.003), but not with the WOMAC scales. Future orientation did not correlate significantly with any outcome.

### Associations of personality traits with changes in HRQoL and WOMAC scores

Multivariate regression models on outcome change scores are presented in Table 5. For patients with THR, a higher baseline self-efficacy was associated with improvement in the EQ-index (β= 0.0704; *p*= 0.0099), the WOMAC global score (β= 6.6337; *p*= 0.0139), WOMAC functioning (β= 8.2557; *p*= 0.0046), and WOMAC pain (β= 5.9994; *p*= 0.0232). The association between self-efficacy and EQ-VAS was positive, but not significant (β= 3.0619; *p*= 0.3402).

**Table 5:** Regression results for associations of personality traits with changes in EQ-5D, EQ-VAS, and WOMAC scores

	**EQ-5D-5L** **utility index**		**EQ-VAS**		**WOMAC global**		**WOMAC** **function**		**WOMAC** **pain**	
	**β THR**	**β TKR**	**β THR**	**β TKR**	**β THR**	**β TKR**	**β THR**	**β TKR**	**β THR**	**β TKR**
Intercept	0.3174 *	0.6567 **	41.0049 *	27.3463	43.2794 **	39.1233 *	31.2369 *	52.0817 **	52.7320 ***	45.7655 **
Self-efficacy	0.0704 **	− 0.0070	3.0619	5.8252 *	6.6337 *	1.1519	8.2557 **	0.0120	5.9994 *	1.1571
Health-related willingness to take risk	− 0.0027	0.0085	− 1.1304	− 0.6109	0.4557	0.9599	0.1420	1.1277	0.1435	1.0180
Future orientation	− 0.0059	0.0007	− 0.7295	1.5918	− 1.1166	0.8937	− 0.8987	0.7182	− 0.9882	0.9310
Male	0.0340	− 0.0553	5.0454	− 2.9442	8.2014	− 5.5233	7.3096	− 5.6967	10.5608 *	1.1747
Age	0.0032	− 0.0017	0.5271	0.0653	0.3433	− 0.2404	0.3184	− 0.2951	0.4970	− 0.1769
Diseases = 1	0.1217	− 0.0105	− 1.5850	1.9280	− 1.2393	10.1159	5.1482	6.0711	− 5.1682	5.1092
= 2	0.0498	− 0.0354	− 2.0378	− 6.9599	− 0.6789	10.6210	3.5706	6.2477	− 4.9668	7.4529
= 3	0.1185	− 0.0181	− 3.2699	− 7.8033	− 2.7762	10.8547	3.9172	5.4063	− 14.7960	5.7977
>= 4	0.0377	− 0.1509	− 6.2499	− 5.1642	− 8.2861	− 7.3776	− 3.7319	− 11.5961	− 11.7986	− 8.4547
Years of Education = 10–11	0.0508	0.0127	17.3616 *	7.9742	NA^a^	NA^a^	NA^a^	NA^a^	NA^a^	NA^a^
= 12–13	− 0.1867 *	− 0.0132	− 7.5909	− 2.3857	NA^a^	NA^a^	NA^a^	NA^a^	NA^a^	NA^a^
> 13	0.0050	− 0.0590	6.5668	− 6.1218	NA^a^	NA^a^	NA^a^	NA^a^	NA^a^	NA^a^
Outcome at baseline	− 0.6667 ***	− 0.6629 ***	− 0.7208 ***	− 0.7337 ***	− 0.8830 ***	− 0.7158 ***	− 0.7998 ***	− 0.7393 ***	− 0.8515 ***	− 0.7912 ***

Each column shows the influence of all included independent variables on the change score of one specific outcome measure (follow-up - baseline)

*THR* Total hip replacement, *TKR* Total knee replacement, *EQ-5D-5L* EuroQol Five-Dimensional Five-Level Questionnaire, *EQ-VAS* EuroQol visual analogue scale, *WOMAC* Western Ontario and McMaster Universities Osteoarthritis Index, general self-efficacy (1–5) higher scores indicate higher self-efficacy, health-related willingness to take risk (0–10) lower values represent risk aversion, future orientation (0–10) higher values indicate a stronger orientation on the future, male 0 = female, 1 = male, diseases present at baseline (0–4) number of diseases present at baseline assessment with 4 aggregating counts of 4 diseases and above, outcome at baseline adjustment for baseline value of the specific outcome

^a^: In estimations with WOMAC as the outcome, education was not part of the minimal sufficient adjustment set, not included as a covariate, and thus appears as NA in the table

Significance: *p < 0.05, **p < 0.01, ***p < 0.001; in bold if the result was at least significant at *p* < 0.05 level

For TKR, only the association of self-efficacy with the EQ-VAS change-score was statistically significant (β= 5.8252; *p*= 0.0482). Self-efficacy demonstrated weak positive, but not significant associations with the WOMAC global score (β= 1.1519; *p*= 0.6998), WOMAC functioning (β= 0.0120; *p*= 0.9972), and WOMAC pain (β= 1.1571; *p*= 0.6843). The association between self-efficacy and EQ-index was close to zero (β=− 0.0070; *p*= 0.8465).

When comparing these changes with the respective MIC values, the associations between self-efficacy and the WOMAC global (β= 6.6337; MIC= 5.87), and WOMAC functioning (β= 8.2557; MIC= 5.96) in the THR cohort exceeded the defined thresholds. The results for TKR overall and other combinations of associations between personality traits and outcomes for THR remained below their respective MIC thresholds.

Supplementary File 4 - Figure S4 depicts the linear associations between self-efficacy and EQ-Index, EQ-VAS, and WOMAC global separately for THR/TKR.

The supplementary files 5 and 6 include the figures S5 and S6 which show linear associations between H-WTTR and future orientation and the outcomes.

H-WTTR was positively, but not significantly associated with all WOMAC scores in THR/TKR patients. Associations with HRQoL outcomes showed no clear positive or negative tendency.

No associations between future orientation and outcome scores were significant. Whereas strictly negative for THR, they were positive for TKR.

Good baseline scores of all outcome measures were associated with smaller improvement in these scores.

### Sensitivity analyses

Replacing future orientation with present orientation led to minor changes in significances and effect sizes (Supplementary File 7 - Table S3). Nevertheless, the implications remained consistent, indicating the robustness of the main analyses.

Neither the linear modeled regression nor the scatter plot implies a distinctive trend of ASA scores over time (Supplementary File 8 - Figure S7). This suggests that the SARS-Cov- 2 pandemic did not create selection bias.

## Discussion

This study provides comprehensive insights into personality traits and their association with outcomes three months after THR/TKR. In patients undergoing THR, higher self-efficacy was associated statistically significant improvements in general OA-specific health status and physical functioning, surpassing the threshold for MIC. Associations of self-efficacy with HRQoL and pain after THR were also statistically significant, but below threshold for MIC.

In contrast, for TKR, higher self-efficacy was associated with improvements in HRQoL; however, these changes did not reach the threshold for MIC. Neither H-WTTR nor future orientation were associated with outcomes after THR or TKR.

Personality traits were also associated with preoperative health status. In THR/TKR, self-efficacy was associated with better HRQoL, physical functioning, and lower pain. H-WTTR was associated with better HRQoL, but not with better functioning or pain. Future orientation was only associated with better HRQoL and physical functioning in THR patients.

### Comparison of the results on self-efficacy with previous research

In THR patients, the association between self-efficacy and improved WOMAC scores corresponds with findings by Brembo et al. [22]. In a secondary analysis of 223 Norwegian patients assessed in 2003 - 2004, they surveyed the impact of social support and self-efficacy, measured by the General Self-Efficacy scale, on the WOMAC global score three months after THR. The significant but weaker association than in the present study may be attributed to different measures to assess self-efficacy. Cultural differences in German and Norwegian patients or health care [52, 53] or innovations in surgical techniques could also explain these differences.

In TKR, the associations between self-efficacy and improved outcome measures were mainly positive, but mostly not significant. Thus, as with THR, self-efficacy may also be beneficial in TKR, but to a lower extent. Furthermore, the smaller improvements in TKR patients may partly explain the lower association and lack of significance between self-efficacy and the outcome.

The non-significant association of self-efficacy with functioning after TKR is comparable to findings by Hartley et al. [23]. Surveying 62 patients on the effect of hope and self-efficacy, on the Self-Efficacy for Rehabilitation Outcome Scale, they observed no significant association with functioning six weeks after THR/TKR. The lack of significance in that study may be due to the short follow-up and the inclusion of patients with TKR with smaller improvements.

However, Hartley et al. [23] also detected an association between self-efficacy and better functioning in patients before undergoing THR/TKR. The same association was found in studies of non-surgical OA patients [54–56]. This suggests that self-efficacy is generally associated with better functioning in patients with OA, regardless of whether they decide to undergo surgery or not.

The MobilE-TRA 2 subproject on patients with vertigo, dizziness, or balance disorders also examined the associations between self-efficacy and treatment outcomes [29]. Consistent with the findings from the OA subproject, higher self-efficacy was associated with treatment success, while H-WTTR and future orientation showed no significant associations. These results suggest that self-efficacy could be a determinant for better treatment outcomes for several conditions that lead to mobility impairment in later life.

### Comparison of the results for H-WTTR and future orientation with previous research

This is the first clinical study assessing the association between H-WTTR, future orientation, and outcomes after THR/TKR and controlling for these two characteristics in a single model with self-efficacy. Notably, H-WTTR was thoroughly validated as an instrument in a large general population study in Germany n≈22,000) [21]. Even though the instrument has not yet been applied in a cohort of THR/TKR patients, the extensive sample size of the validation study and the emphasis on representative results with sufficient statistical power support its validity and transferability to various clinical populations [21].

The absence of associations between H-WTTR or future orientation and outcomes contrasts with initial expectations. Due to the results of previous research linking lower H-WTTR and higher future orientation to better adherence, we expected a positive influence on the outcome [21, 25, 57]. The lack of association between H-WTTR and outcome at follow-up might be explained by patients with higher H-WTTR scores surpassing the recommended workload, potentially accelerating functional improvement in some cases. This may compensate for possible health risks due to lower compliance, thereby preventing a deterioration in functioning. In addition, due to the short observation period characterized by intensive postoperative supervision, behavioral consequences of H-WTTR and future orientation on the outcome might have been mitigated. Furthermore, the assessed concept of future orientation may capture a more distant future.

### Further interpretation of results

As expected, the mobility and pain dimensions of the EQ-5D-5L improved the most, as these are the most commonly affected domains in patients with hip and knee OA [58]. Notably, THR was not only associated with improvements in physical function but also with reductions in anxiety and depression, suggesting potential additional benefits of the intervention.

Focusing on preoperative outcomes, the positive association between H-WTTR and HRQoL may reflect a potential link between risk-taking health behaviors and a greater willingness to accept the potential risks of surgery. Consequently, patients with higher H-WTTR might have decided to undergo surgery at an earlier stage of their disease when their quality of life was still relatively less impaired compared to individuals with lower H-WTTR scores.

Similarly, the positive association between future orientation and baseline outcomes in THR suggests that a stronger focus on long-term health improvements may influence the timing of surgery. More future-oriented patients might decide to undergo THR earlier despite initial discomfort or less severe impairment. The predominantly positive associations between future orientation and outcomes before TKR point to a comparable decision-making.

Higher baseline scores in functioning and quality of life were strongly associated with less improvement, which is a well-known phenomenon in studies on prognostic factors for outcomes following THR/TKR [6, 59]. These associations may result from a ceiling effect of patient-assessed outcomes in THR/TKR [52].

### Strengths and Limitations

A key strength of this study is its design based on primary data, whereas comparable previous studies relied on secondary data [22] or a smaller sample size [23]. Furthermore, the differentiation between THR and TKR revealed distinct sample-specific results. High participation and follow-up rates, a high level of data completeness, and a careful and well-informed development of the regression model further underline this study’s robustness and methodological quality.

However, some limitations should be acknowledged. First, the monocentric design at a university hospital may limit the generalizability of the findings to other healthcare settings. Second, the three-month follow-up period might have been too short to fully capture potential associations between H-WTTR or future orientation and long-term health outcomes. Third, the applied self-reported measures may have been subject to self-report bias. Despite the selection of validated measures, self-report bias could not be completely excluded.

## Conclusion

This study clarifies how selected personality traits are associated with the outcome in HRQoL and functioning after THR/TKR. Further research is necessary to confirm the prognostic properties of self-efficacy. If confirmed, pre-operative strategies that foster self-efficacy should be integrated into prehabilitation programs [24, 60–62]. Our findings do not support the implementation of H-WTTR and future orientation interventions before THR/TKR. However, these traits might be relevant for long-term treatment success. Subsequent studies should consider extended follow-up periods.

## Supplementary Information

Supplementary Material 1

Supplementary Material 2

Supplementary Material 3

Supplementary Material 4

Supplementary Material 5

Supplementary Material 6

Supplementary Material 7

Supplementary Material 8

## Data Availability

Data is available upon reasonable request. The collected data cannot be shared publicly because the patients did not consent to the deposit of their data in a public repository. Access to any individual-level patient data is not available.
