# Supplementary material for: Association of self-efficacy, risk attitudes, and time preferences with health-related quality of life and functioning after total hip or knee replacement – Results of the MobilE-TRA 2 cohort
Source: Health Qual Life Outcomes. 2025 Apr 23;23:44. doi: 10.1186/s12955-025-02374-y (PMC12020169; doi:10.1186/s12955-025-02374-y)
Supplement: Supplementary file 1 — Supplementary Material 1 [file 12955_2025_2374_MOESM1_ESM.docx]

**Supplementary File 1: Table S1:** List of chronic diseases provided in the questionnaire.

The following prespecified options were given to state diseases in the questionnaire:

- Asthma
- COPD (Chronic obstructive pneumonia disease) or emphysema
- Chronic bronchitis
- Neurologic disease (multiple sclerosis or epilepsy)
- Liver disease (e.g. cirrhosis)
- Heart failure
- Insufficient coronal blood supply (angina pectoris, coronary artery disease)
- Arrhythmia
- Hay fever
- Kidney disease
- Inflammatory joint disease (explicitly not osteoarthritis)
- Hypertension
- Other diseases (cold explicitly excluded)
