# Supplementary material for: Association of self-efficacy, risk attitudes, and time preferences with health-related quality of life and functioning after total hip or knee replacement – Results of the MobilE-TRA 2 cohort
Source: Health Qual Life Outcomes. 2025 Apr 23;23:44. doi: 10.1186/s12955-025-02374-y (PMC12020169; doi:10.1186/s12955-025-02374-y)
Supplement: Supplementary file 2 — Supplementary Material 2 [file 12955_2025_2374_MOESM2_ESM.docx]

**Supplementary File 2: DAG Models**

**Supplementary File 2: Figure S1:** HRQoL model


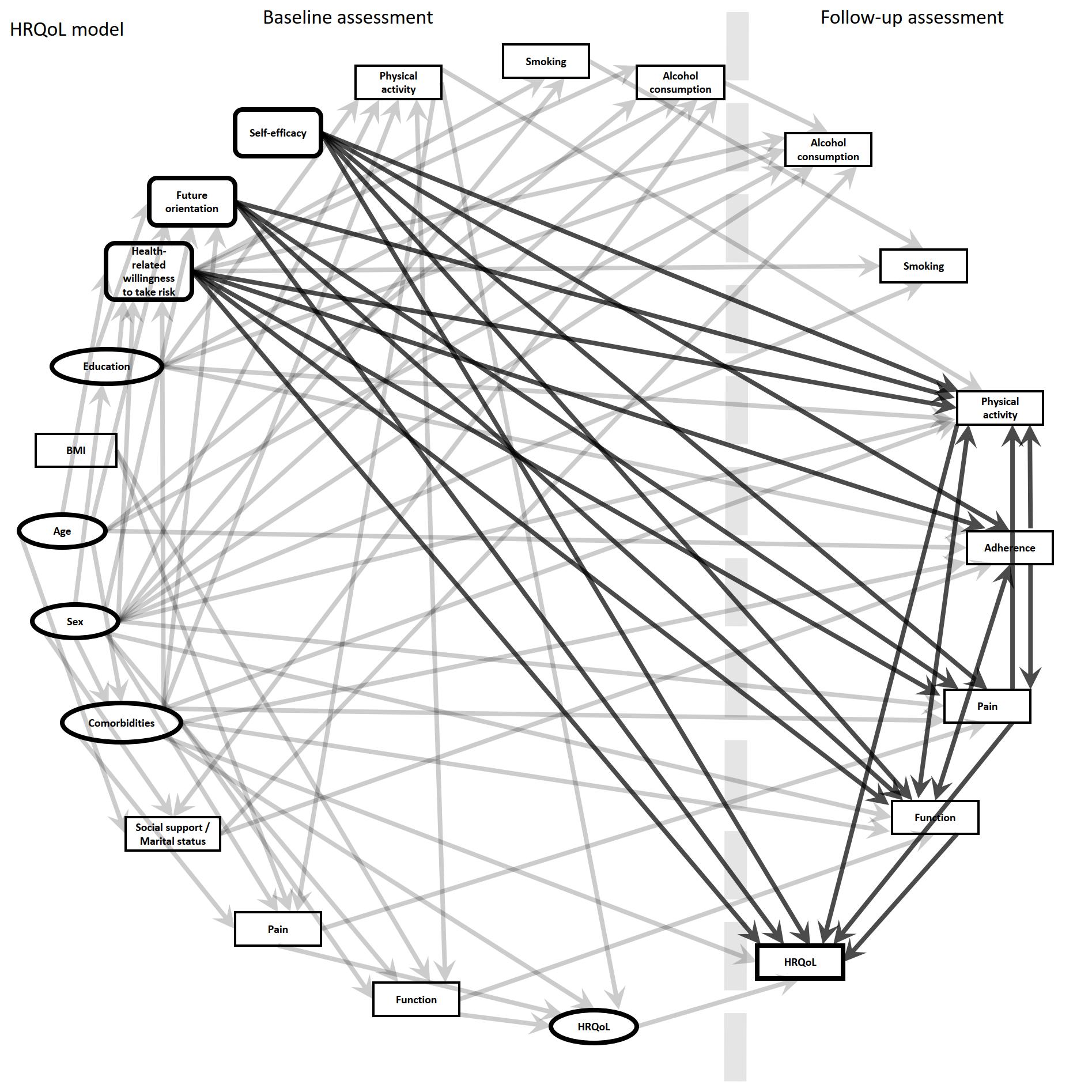


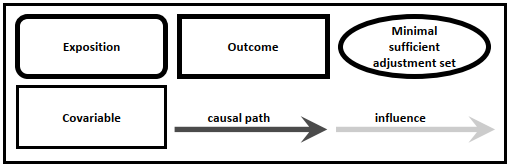


**Supplementary File 2: Figure S2:** Function model


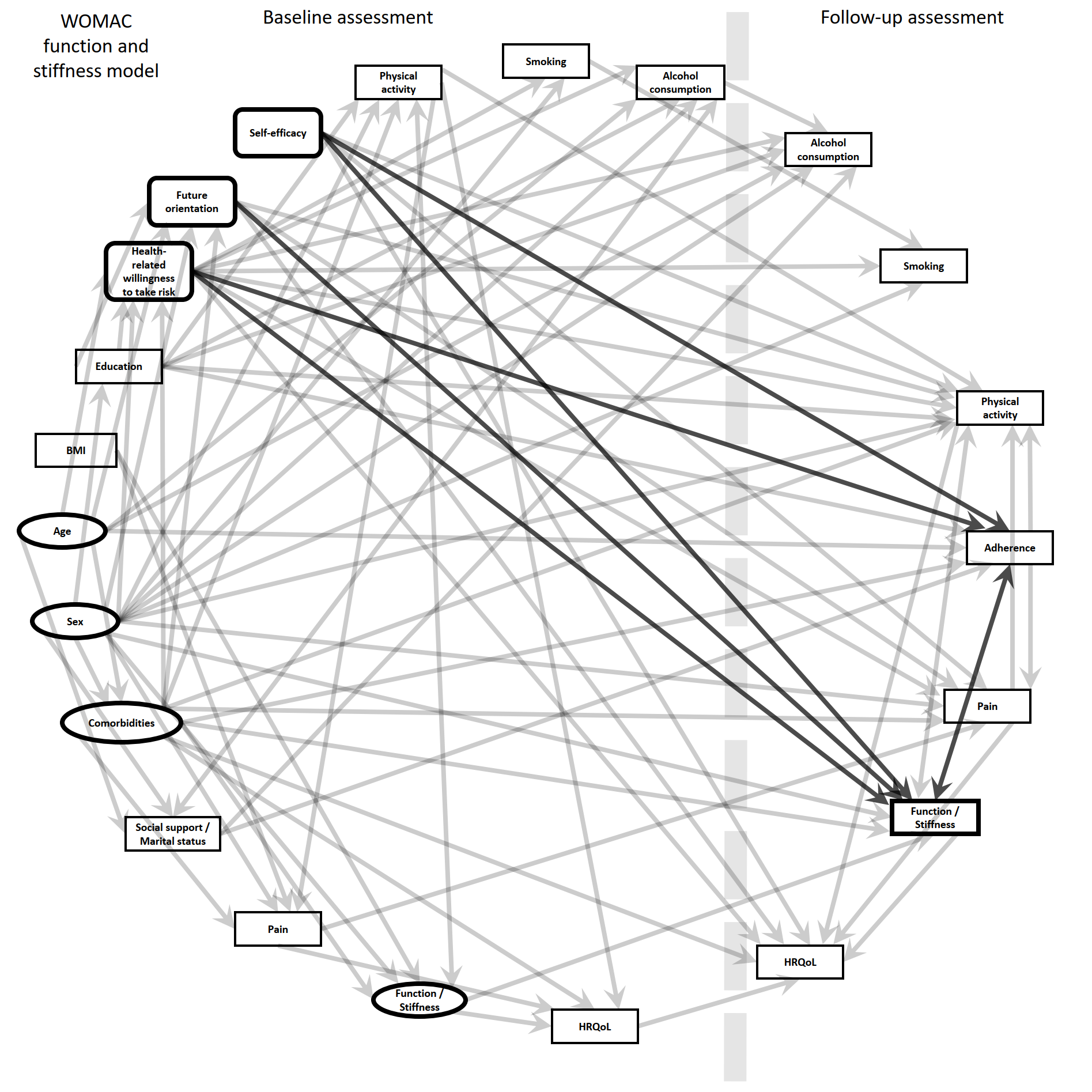

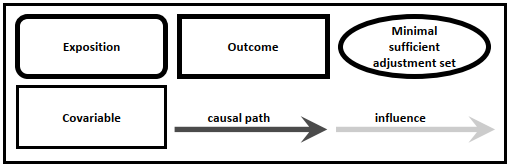


**Supplementary File 2: Figure S3:** Pain model


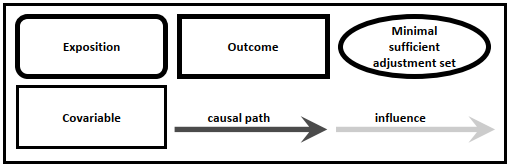

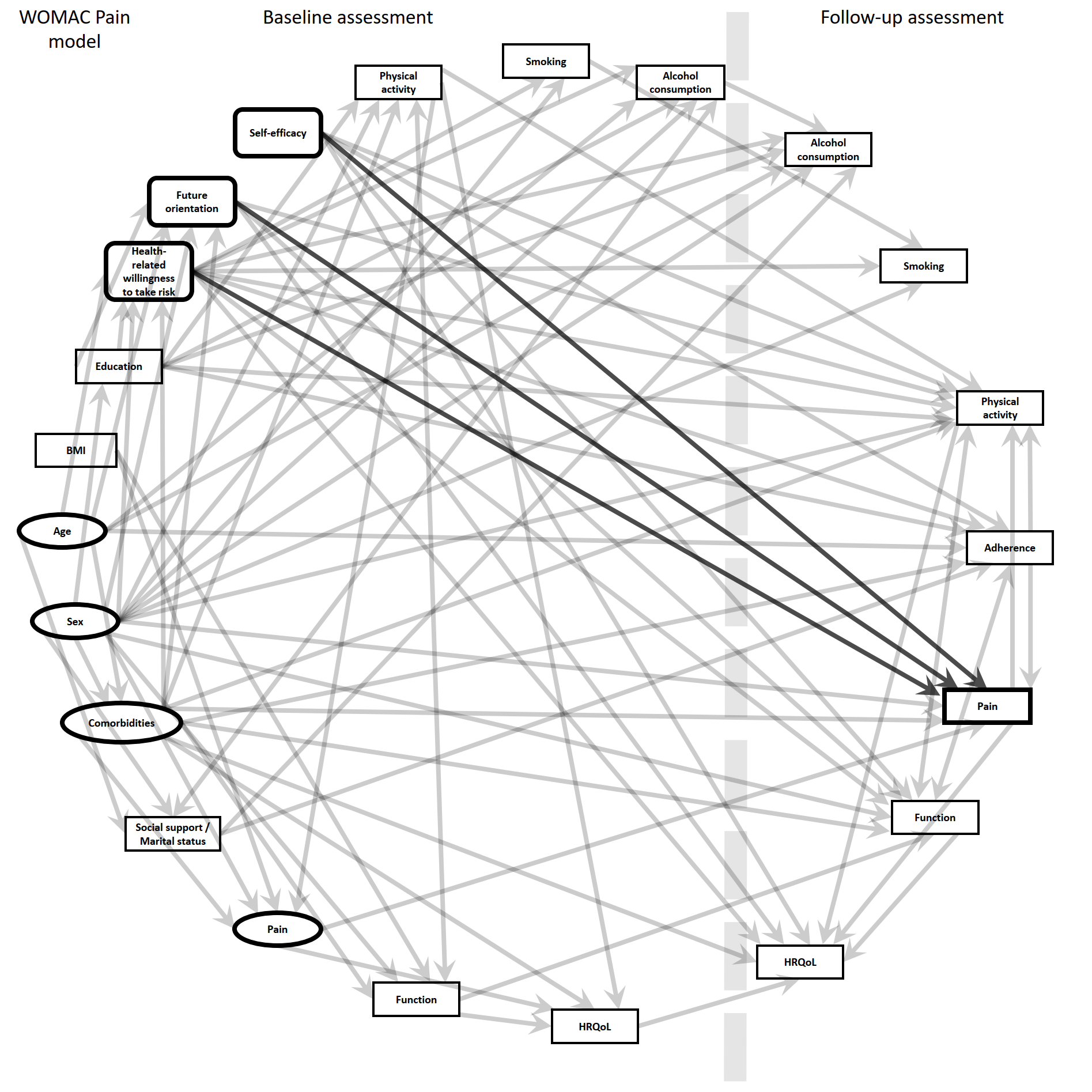


References

1. Acree LS, Longfors J, Fjeldstad AS, Fjeldstad C, Schank B, Nickel KJ, et al. Physical activity is related to quality of life in older adults. Health Qual Life Out. 2006;4(1):37.

2. Allen K, Morey M. Physical Activity and Adherence. 2010. p. 9-38.

3. Anderson LR, Mellor JM. Predicting health behaviors with an experimental measure of risk preference. J Health Econ. 2008;27(5):1260-74.

4. Andrew JG, Palan J, Kurup HV, Gibson P, Murray DW, Beard DJ. Obesity in total hip replacement. J Bone Joint Surg Br. 2008;90-B(4):424-9.

5. Bauer T, Göhlmann S, Sinning M. Gender differences in smoking behavior. Health Econ. 2007;16(9):895-909.

6. Bradford WD. The association between individual time preferences and health maintenance habits. Med Decis Making. 2010;30(1):99-112.

7. Brembo EA, Kapstad H, Van Dulmen S, Eide H. Role of self-efficacy and social support in short-term recovery after total hip replacement: a prospective cohort study. Health Qual Life Out. 2017;15(1):68.

8. Brettschneider C, Leicht H, Bickel H, Dahlhaus A, Fuchs A, Gensichen J, et al. Relative impact of multimorbid chronic conditions on health-related quality of life - Osteoarthritis and Cartilageresults from the MultiCare Cohort Study. PLoS One. 2013;8(6):e66742.

9. Cesari M, Onder G, Russo A, Zamboni V, Barillaro C, Ferrucci L, et al. Comorbidity and Physical Function: Results from the Aging and Longevity Study in the Sirente Geographic Area (ilSIRENTE Study). Gerontology. 2006;52(1):24-32.

10. Daugaard R, Tjur M, Sliepen M, Lipperts M, Grimm B, Mechlenburg I. Are patients with knee osteoarthritis and patients with knee joint replacement as physically active as healthy persons? J Orthop Transl. 2018;14:8-15.

11. Decker S, Schmitz H. Health shocks and risk aversion. J Health Econ. 2016;50:156-70.

12. Dohmen T, Falk A, Huffman D, Sunde U, Schupp J, Wagner GG. Individual risk attitudes: Measurement, determinants, and behavioral consequences. J Eur Econ Assoc. 2011;9(3):522-50.

13. Garnett C, Kastaun S, Brown J, Kotz D. Alcohol consumption and associations with sociodemographic and health-related characteristics in Germany: A population survey. Addict Behav. 2022;125:107159.

14. Gast A, Mathes T. Medication adherence influencing factors—an (updated) overview of systematic reviews. Syst Rev-London. 2019;8(1):112.

15. Hajek A, Bock J-O, Weyerer S, König H-H. Correlates of alcohol consumption among Germans in the second half of life. Results of a population-based observational study. BMC Geriatr. 2017;17(1):207.

16. Hawke LJ, Shields N, Dowsey MM, Choong PFM, Taylor NF. Physical activity levels after hip and knee joint replacement surgery: an observational study. Clin Rheumatol. 2019;38(3):665-74.

17. Hiller J, Schatz K, Drexler H. Gender influence on health and risk behavior in primary prevention: a systematic review. J Public Health. 2017;25(4):339-49.

18. Hurley M, Dickson K, Hallett R, Grant R, Hauari H, Walsh N, et al. Exercise interventions and patient beliefs for people with hip, knee or hip and knee osteoarthritis: a mixed methods review. Cochrane Database Syst Rev. 2018(4).

19. Jakobsen JC, Gluud C, Wetterslev J, Winkel P. When and how should multiple imputation be used for handling missing data in randomised clinical trials – a practical guide with flowcharts. BMC Med Res Methodol. 2017;17(1):162.

20. Kelly S, Olanrewaju O, Cowan A, Brayne C, Lafortune L. Alcohol and older people: A systematic review of barriers, facilitators and context of drinking in older people and implications for intervention design. PLoS One. 2018;13(1):e0191189.

21. Lopes DG, Costa D, Cruz EB, Mendonça N, Henriques AR, Branco J, et al. Association of physical activity with physical function and quality of life in people with hip and knee osteoarthritis: longitudinal analysis of a population-based cohort. Arthritis Res Ther. 2023;25(1):14.

22. McCalden RW, Charron KD, MacDonald SJ, Bourne RB, Naudie DD. Does morbid obesity affect the outcome of total hip replacement? J Bone Joint Surg Br. 2011;93-B(3):321-5.

23. McKevitt S, Healey E, Jinks C, Rathod-Mistry T, Quicke J. The association between comorbidity and physical activity levels in people with osteoarthritis: Secondary analysis from two randomised controlled trials. Osteoarthritis and Cartilage Open. 2020;2(2):100057.

24. Notthoff N, Reisch P, Gerstorf D. Individual characteristics and physical activity in older adults: a systematic review. Gerontology. 2017;63(5):443-59.

25. Pinquart M, Sörensen S. Influences on Loneliness in Older Adults: A Meta-Analysis. Basic Appl Soc Psych. 2001;23:245-66.

26. Pisters MF, Veenhof C, Schellevis FG, Twisk JW, Dekker J, De Bakker DH. Exercise adherence improving long‐term patient outcome in patients with osteoarthritis of the hip and/or knee. Arthritis Care Res. 2010;62(8):1087-94.

27. Rivera-Torres S, Fahey TD, Rivera MA. Adherence to exercise programs in older adults: informative report. Gerontol Geriatr Med. 2019;5:2333721418823604.

28. Rosemann T, Kuehlein T, Laux G, Szecsenyi J. Osteoarthritis of the knee and hip: a comparison of factors associated with physical activity. Clin Rheumatol. 2007;26(11):1811-7.

29. Rueda S, Artazcoz L, Navarro V. Health inequalities among the elderly in western Europe. J Epidemiol Community Health. 2008;62(6):492-8.

30. Ryan A, Wallace E, O’Hara P, Smith SM. Multimorbidity and functional decline in community-dwelling adults: a systematic review. Health Qual Life Out. 2015;13(1):168.

31. Stephan A-J, Schwettmann L, Meisinger C, Ladwig K-H, Linkohr B, Thorand B, et al. Living longer but less healthy: The female disadvantage in health expectancy. Results from the KORA-Age study. Exp Gerontol. 2021;145:111196.

32. Sutter M, Weyland M, Untertrifaller A, Froitzheim M, Schneider SO. Financial Literacy, Experimental Preference Measures and Field Behavior–A Randomized Educational Intervention. Discussion Papers of the Max Planck Institute for Research on Collective Goods. 2023.

33. Van Der Pol M, Hennessy D, Manns B. The role of time and risk preferences in adherence to physician advice on health behavior change. Eur J Health Econ. 2017;18(3):373-86.

34. Weller IMR, Kunz M. Physical activity and pain following total hip arthroplasty. Physiotherapy. 2007;93(1):23-9.
