# Supplementary material for: Association of self-efficacy, risk attitudes, and time preferences with health-related quality of life and functioning after total hip or knee replacement – Results of the MobilE-TRA 2 cohort
Source: Health Qual Life Outcomes. 2025 Apr 23;23:44. doi: 10.1186/s12955-025-02374-y (PMC12020169; doi:10.1186/s12955-025-02374-y)
Supplement: Supplementary file 3 — Supplementary Material 3 [file 12955_2025_2374_MOESM3_ESM.docx]

**Supplementary File 3: Table S2:** Missing values in the final n = 144 cohort.

| Variable | Missing Values | Total number assessed |
| --- | --- | --- |
| EQ-5D-5L utility index | 0 | 288 |
| EQ-VAS | 0 | 288 |
| WOMAC global | 0 | 288 |
| WOMAC function | 0 | 288 |
| WOMAC pain | 0 | 288 |
| General self-efficacy | 0 | 144 |
| Health-related willingness to take risk | 0 | 144 |
| Future orientation | 2 | 144 |
| Sex | 0 | 288 |
| Age | 0 | 288 |
| Education | 0 | 144 |
| Diseases present at baseline | 1 | 3744 ^a^ |
|  |  |  |

EQ-5D-5L: EuroQol Five-Dimensional Five-Level Questionnaire; EQ-VAS: EuroQol visual analogue scale; WOMAC: Western Ontario and McMaster Universities Osteoarthritis Index. ^a^ 2 periods, 13 questions 147 participants: 2*13*144 = 3744
