# Supplementary material for: Association of self-efficacy, risk attitudes, and time preferences with health-related quality of life and functioning after total hip or knee replacement – Results of the MobilE-TRA 2 cohort
Source: Health Qual Life Outcomes. 2025 Apr 23;23:44. doi: 10.1186/s12955-025-02374-y (PMC12020169; doi:10.1186/s12955-025-02374-y)
Supplement: Supplementary file 4 — Supplementary Material 4 [file 12955_2025_2374_MOESM4_ESM.docx]

**Supplementary File 4: Figure S4: Association between self-efficacy and change in each main outcome for THR/TKR.**

| THR | TKR |
| --- | --- |
| 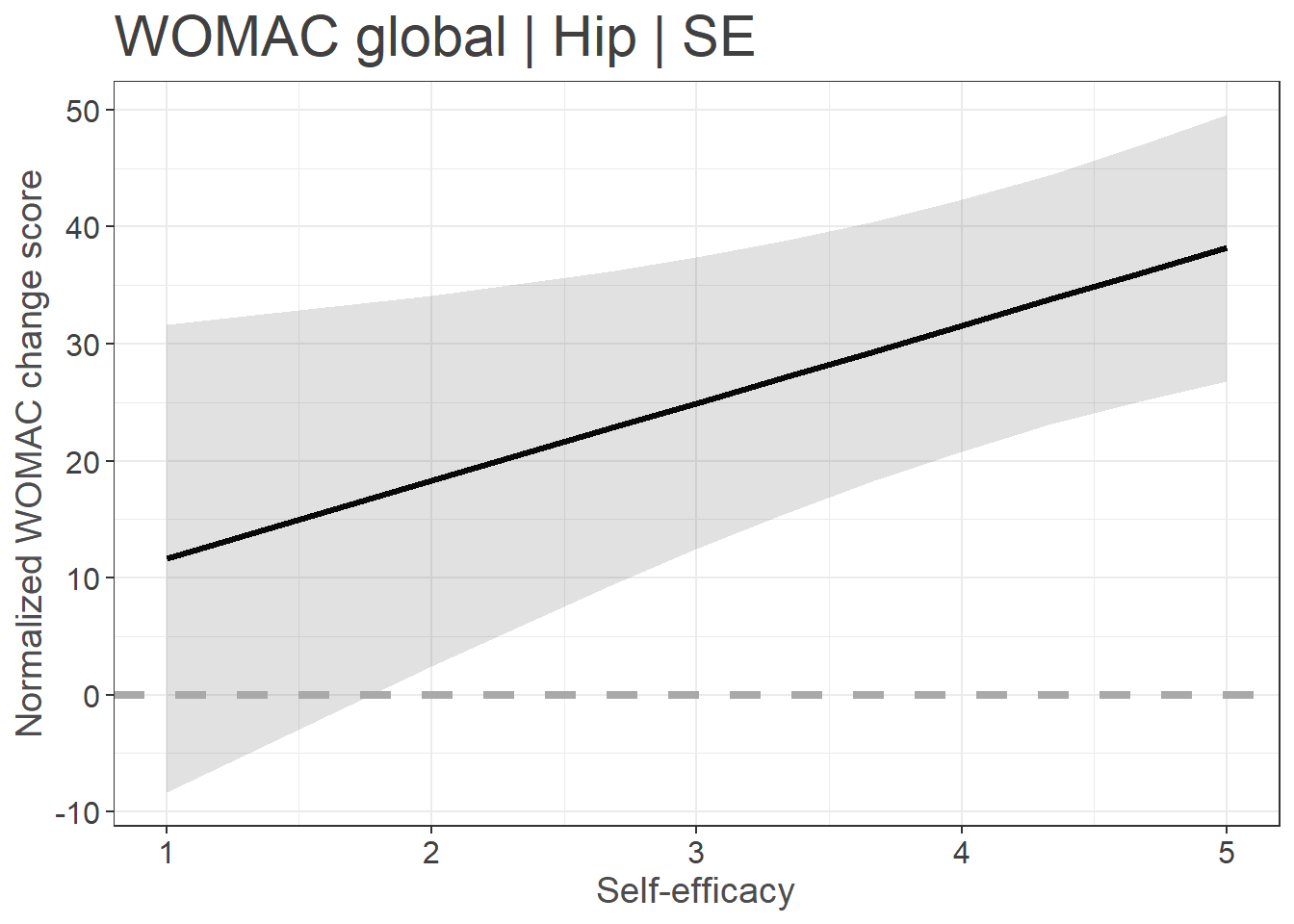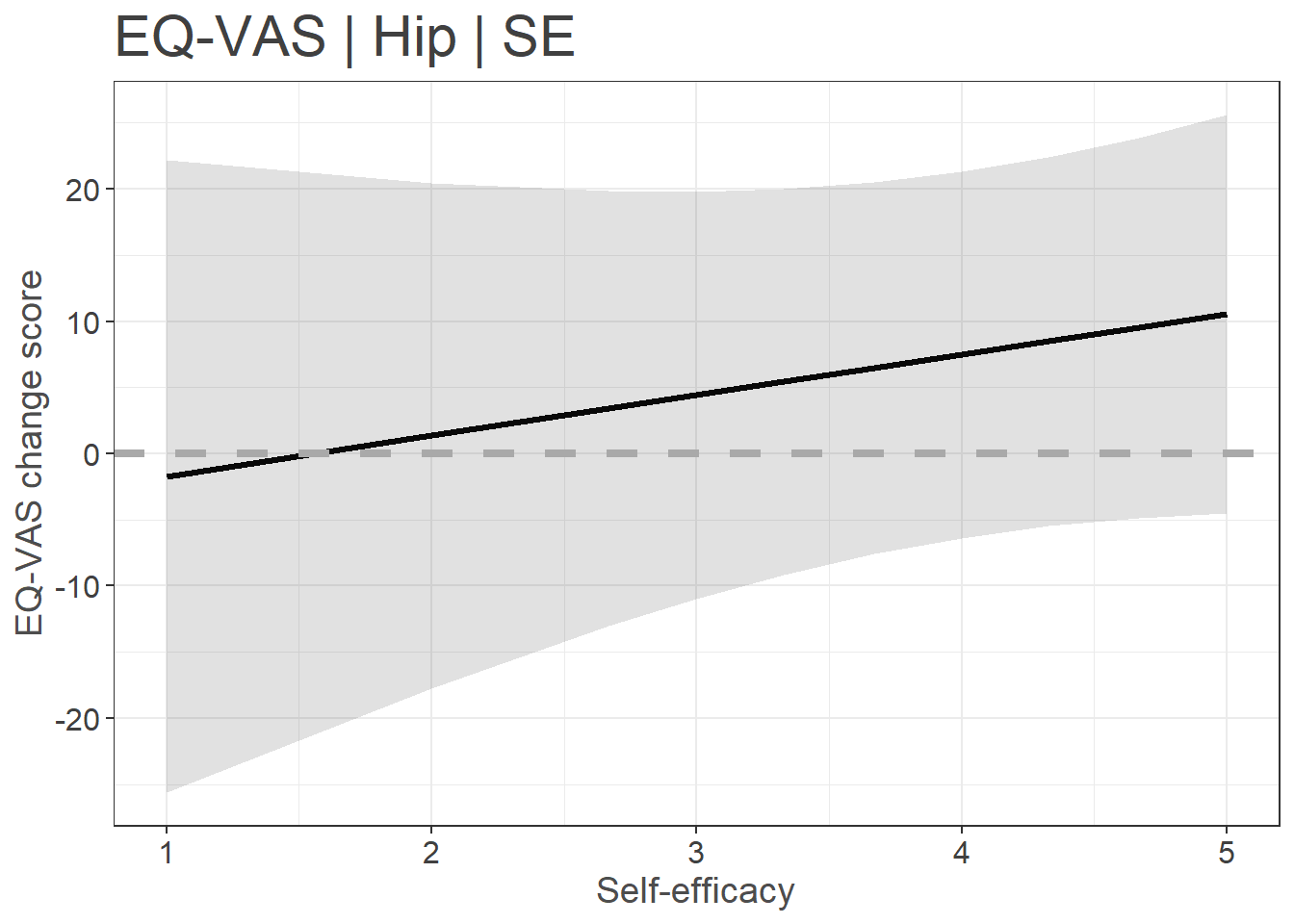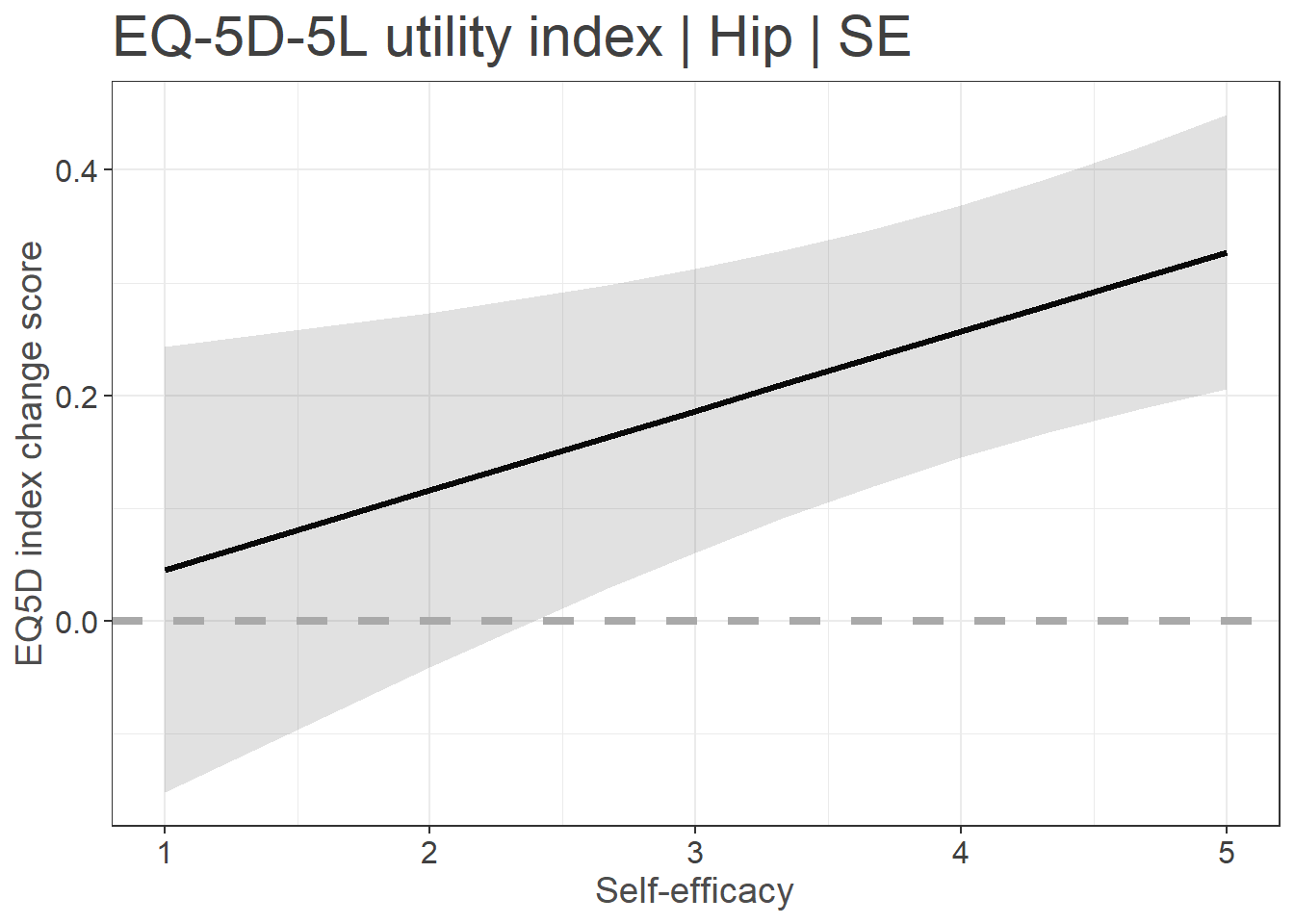 | 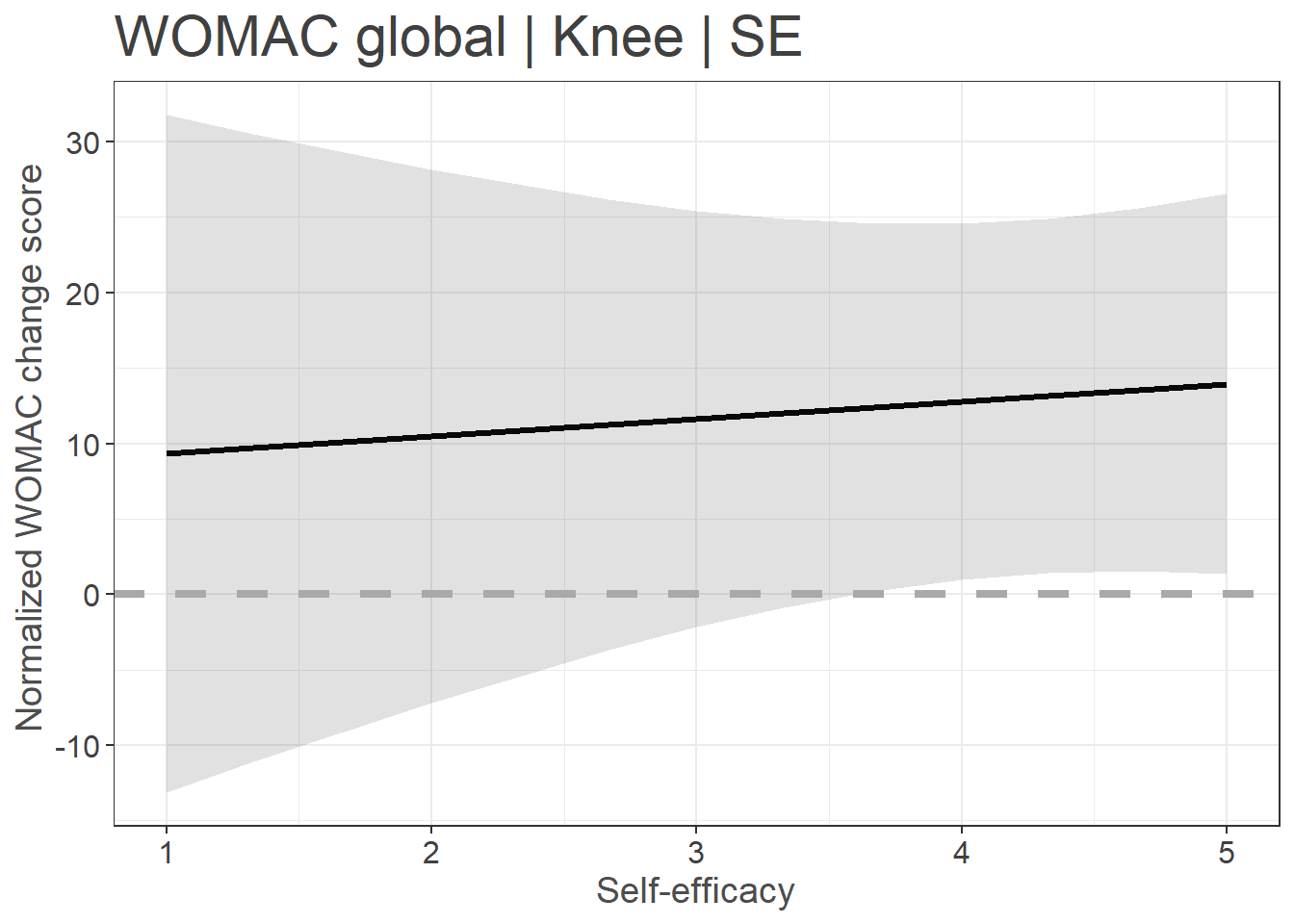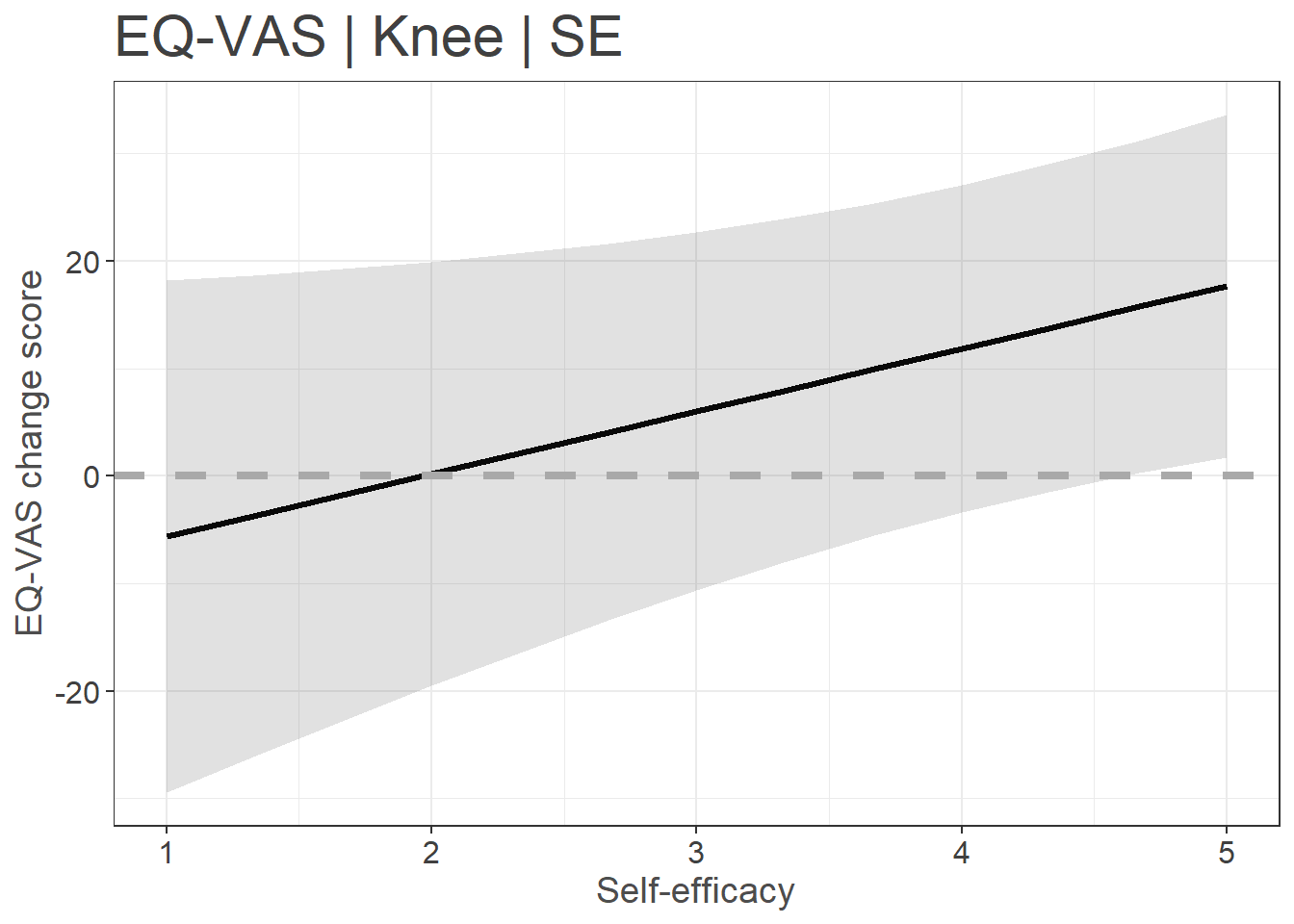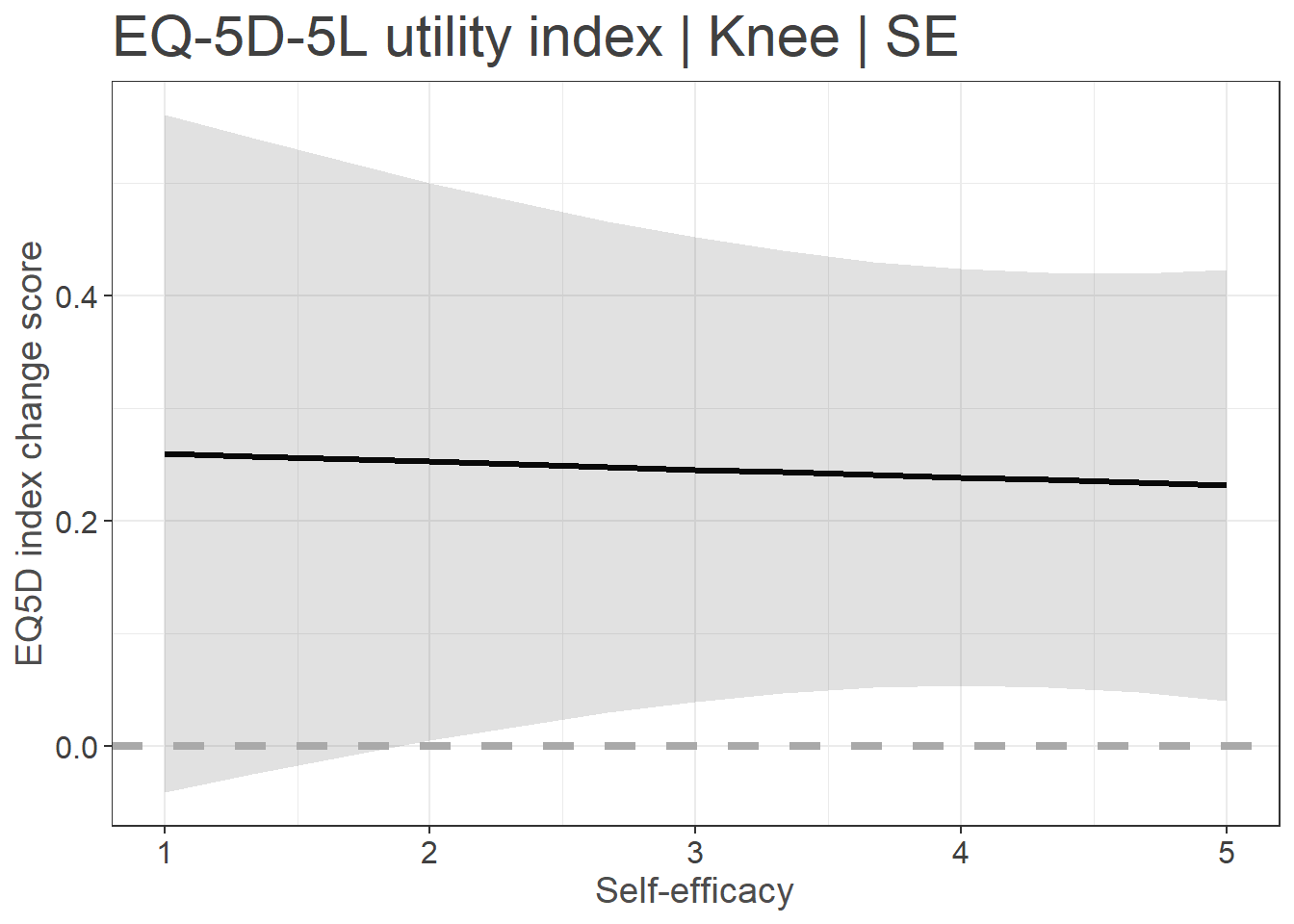 |

EQ-5D-5L: EuroQol Five-Dimensional Five-Level Questionnaire; EQ-VAS: EuroQol visual analogue scale; WOMAC global: Western Ontario and McMaster Universities Osteoarthritis Index global score; SE: general self-efficacy, higher scores indicate higher self-efficacy. Effects plot based on adjusted linear estimation models. The solid black line shows the estimated influence on the change score of the specific outcome measure (follow-up - baseline). The areas surrounding each curve are confidence bands based on a 95% confidence interval. Self-efficacy is positioned on the x-axis and EQ-5D-5L utility index / EQ-VAS (0-100) / WOMAC global score (0-100) on the y-axis. WOMAC score is inverted, higher values represent better health status. All covariates were held constant (mean value for continuous variables; reference level for factors).
