# Supplementary material for: Association of self-efficacy, risk attitudes, and time preferences with health-related quality of life and functioning after total hip or knee replacement – Results of the MobilE-TRA 2 cohort
Source: Health Qual Life Outcomes. 2025 Apr 23;23:44. doi: 10.1186/s12955-025-02374-y (PMC12020169; doi:10.1186/s12955-025-02374-y)
Supplement: Supplementary file 6 — Supplementary Material 6 [file 12955_2025_2374_MOESM6_ESM.docx]

**Supplementary File 6: Figure S6: Association between future orientation and change in each main outcome for THR/TKR.**

| THR | TKR |
| --- | --- |
| 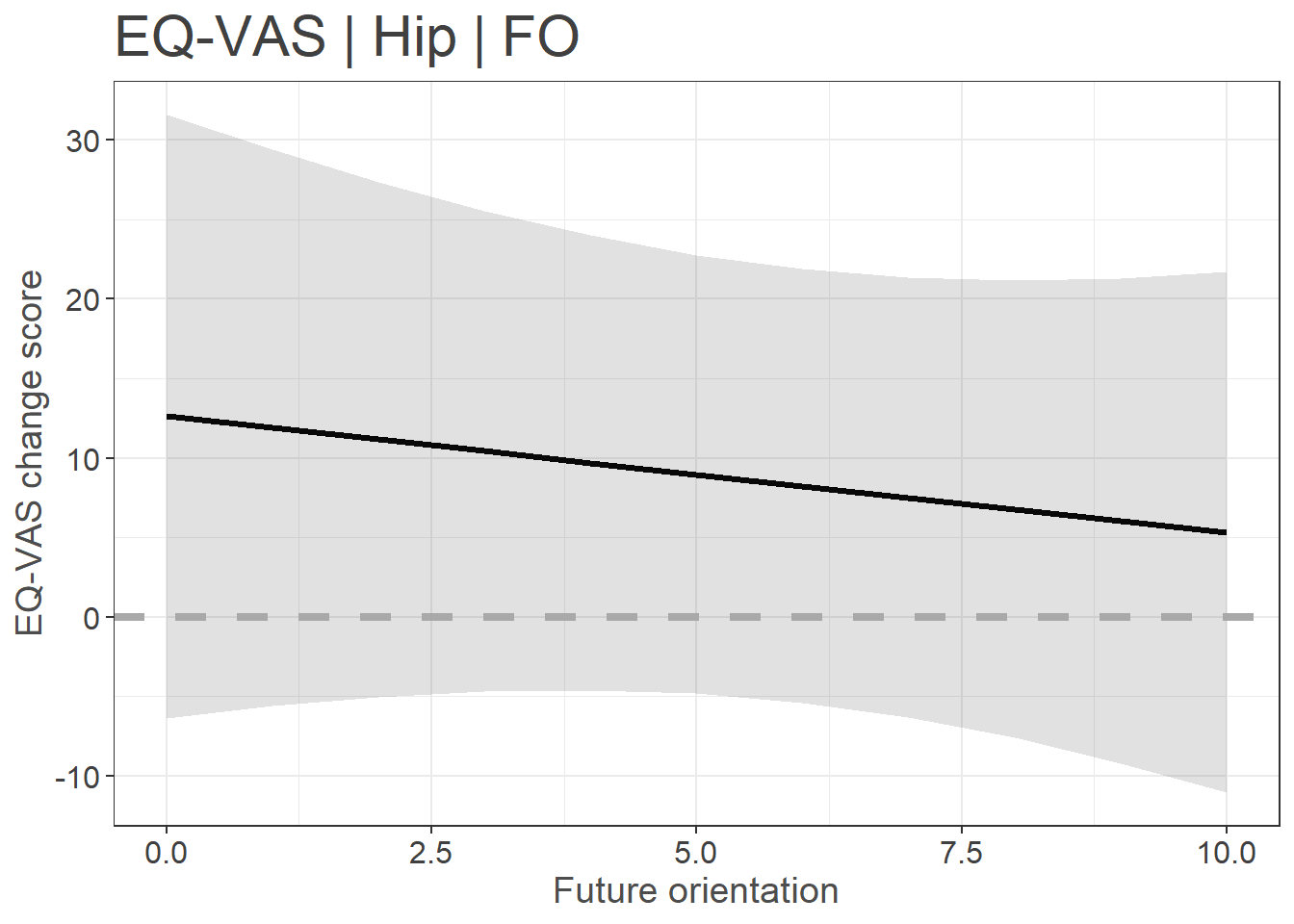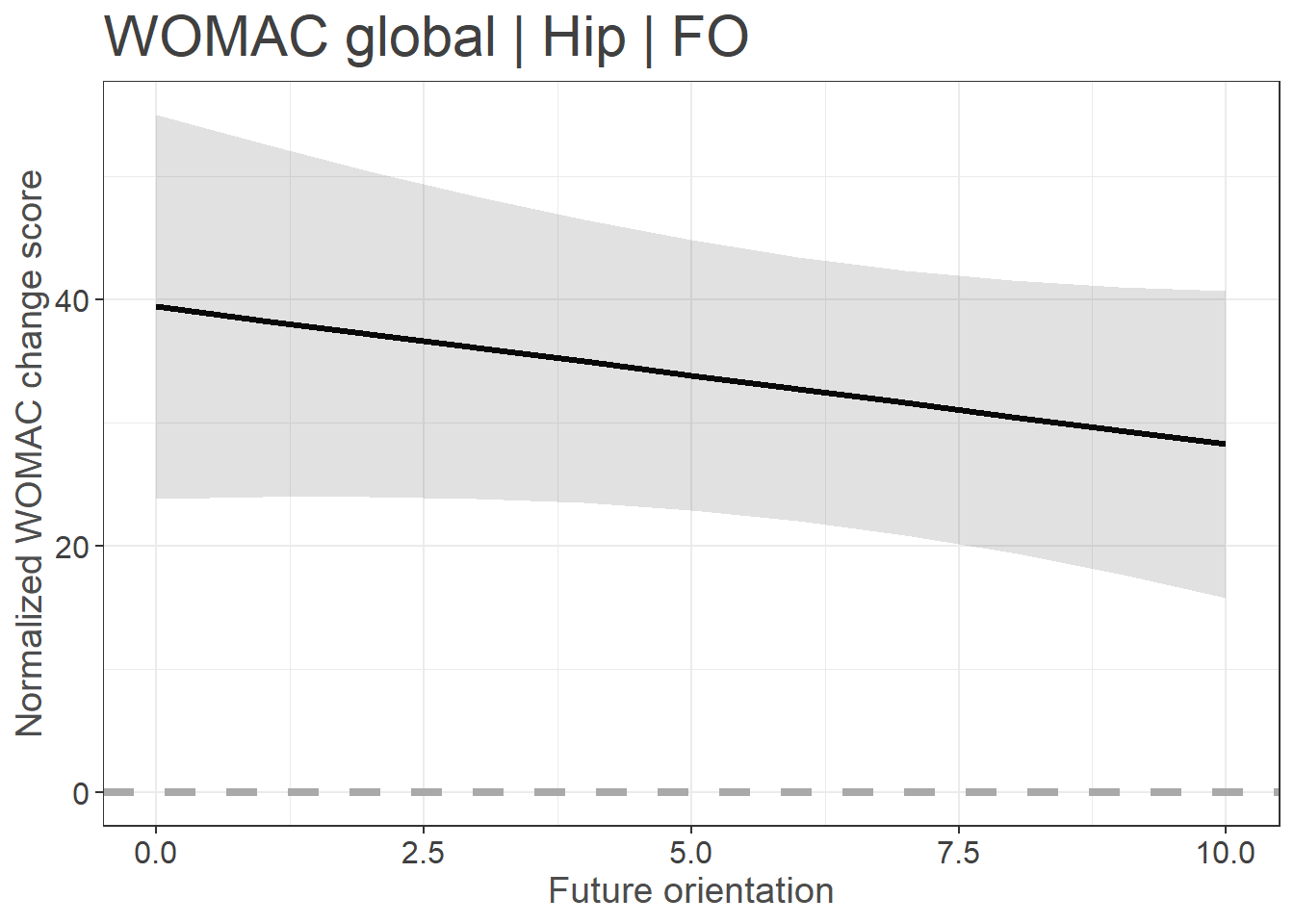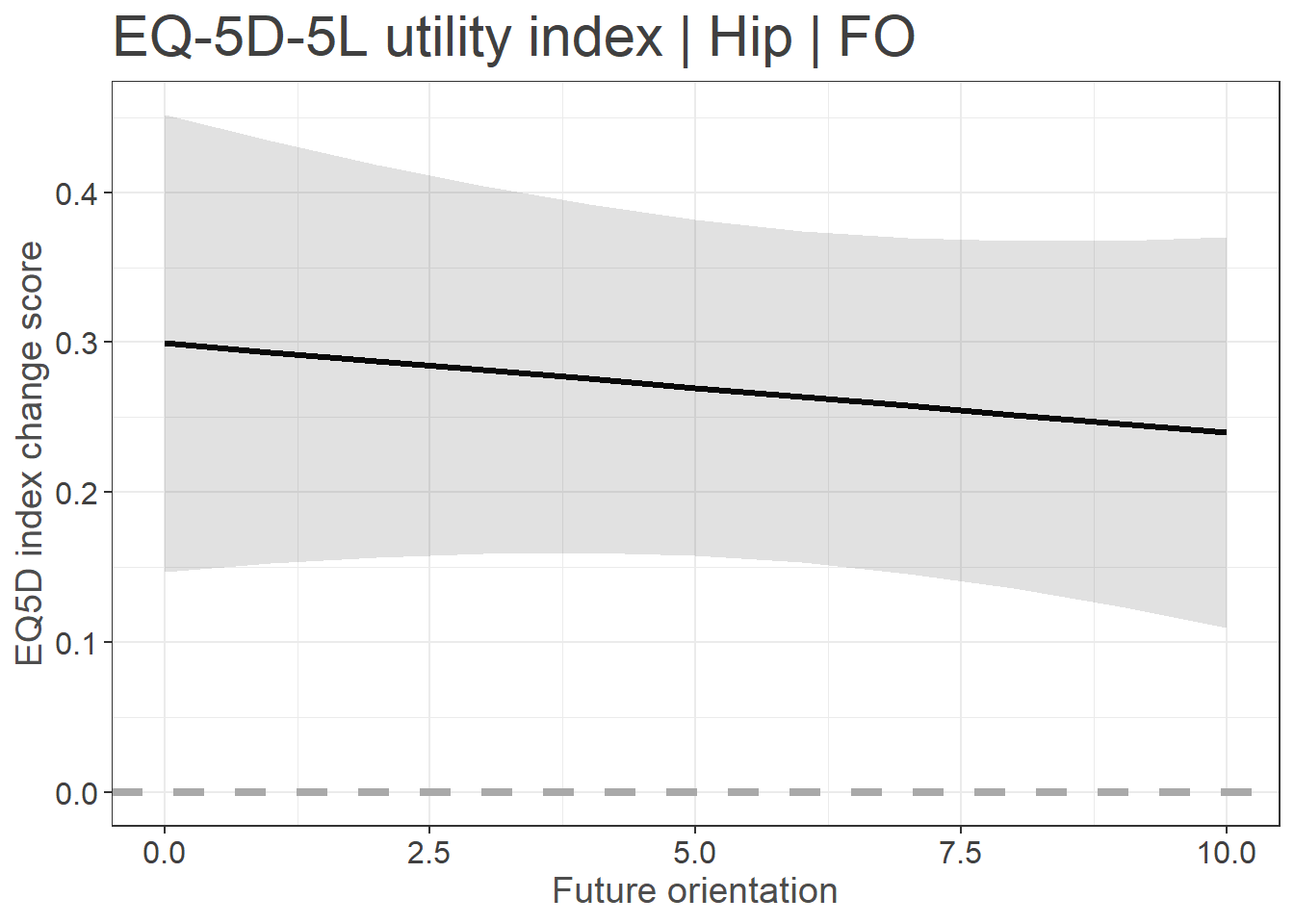 | 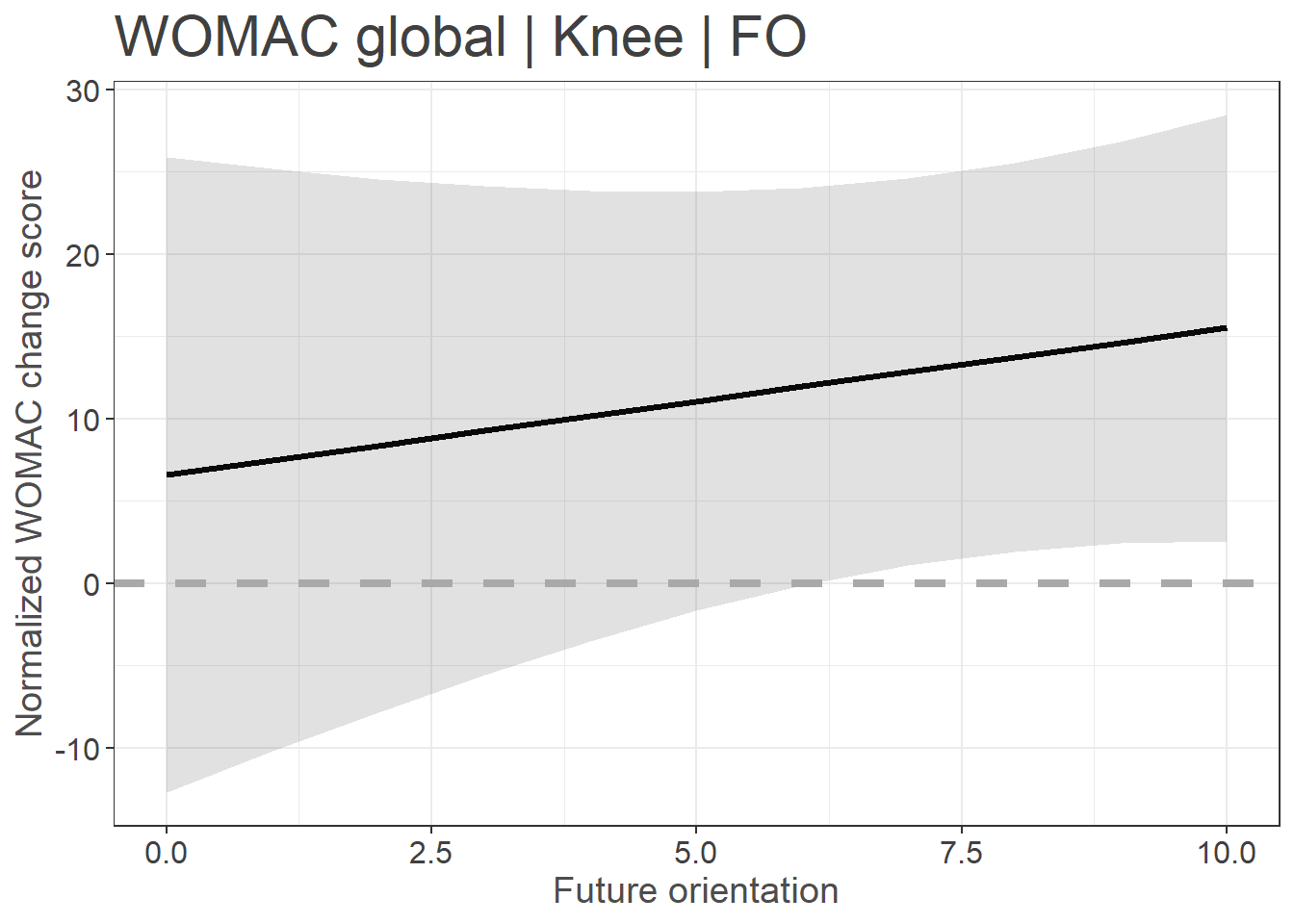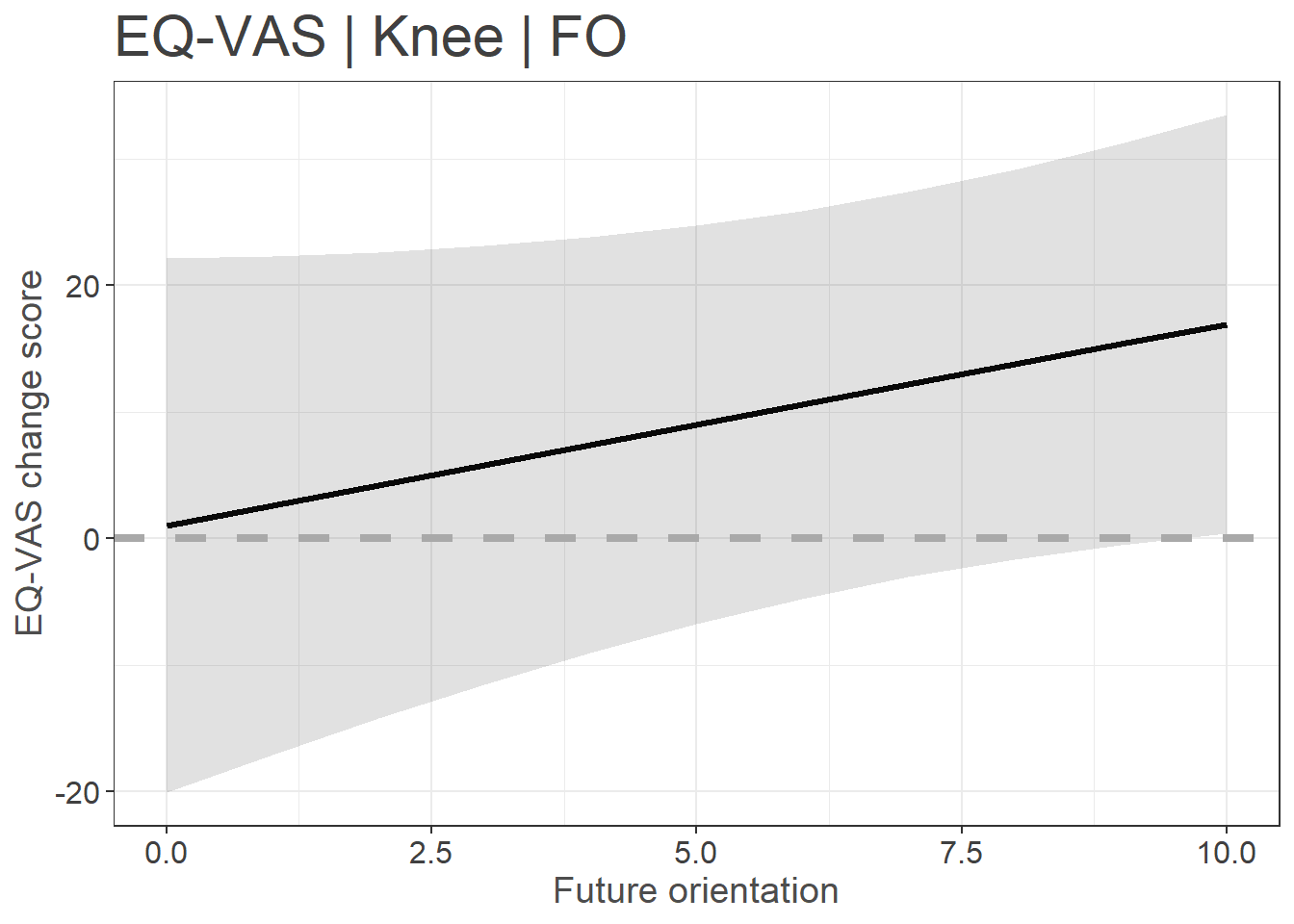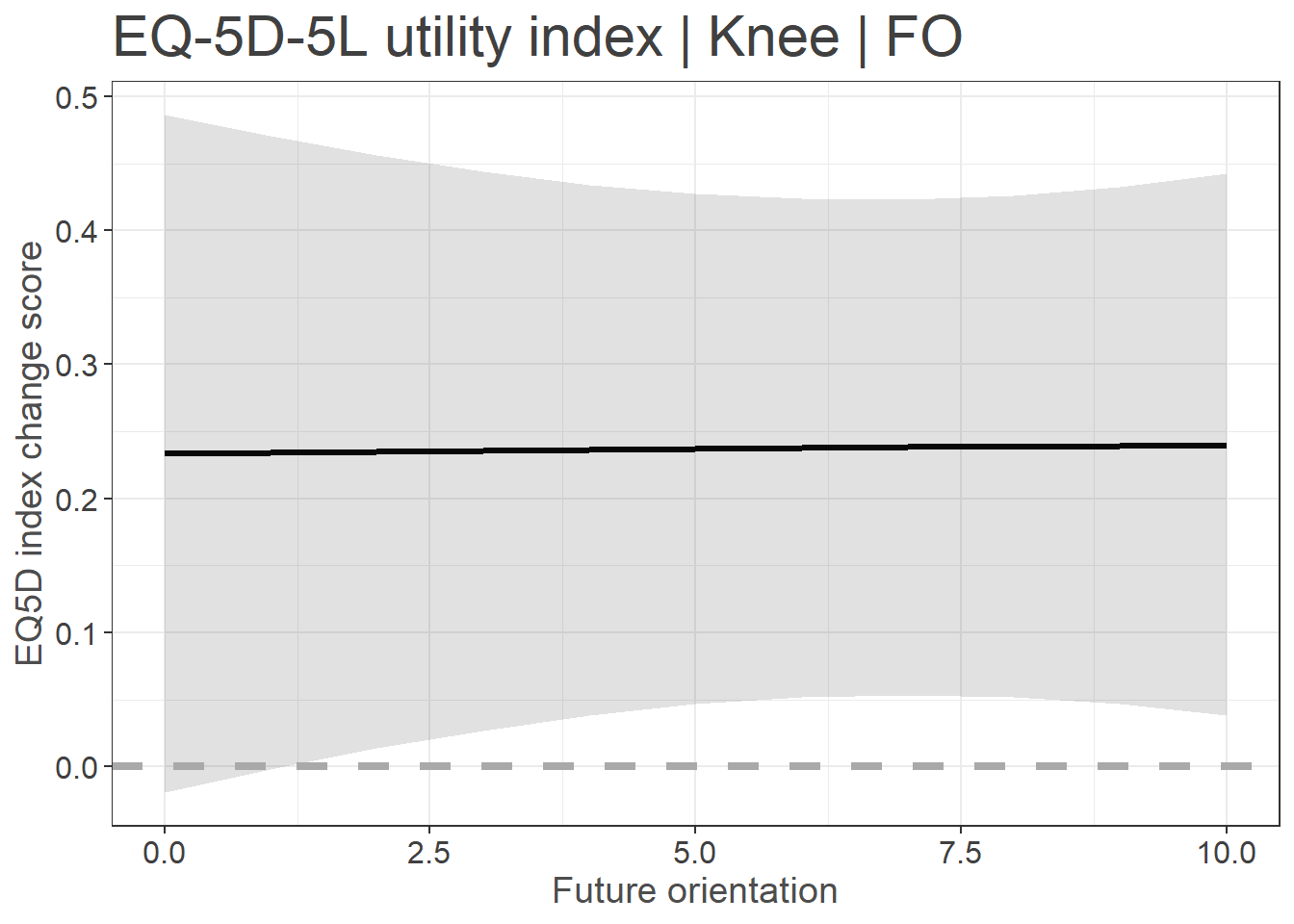 |

EQ-5D-5L: EuroQol Five-Dimensional Five-Level Questionnaire; EQ-VAS: EuroQol visual analogue scale; WOMAC global: Western Ontario and McMaster Universities Osteoarthritis Index global score; FO: future orientation (0-10): higher values indicate a stronger orientation on the future. Effects plot based on adjusted linear estimation models. The solid black line shows the estimated influence on the change score of the specific outcome measure (follow-up - baseline). The areas surrounding each curve are confidence bands based on a 95% confidence interval. FO is positioned on the x-axis and EQ-5D-5L utility index / EQ-VAS (0-100) / WOMAC global score (0-100) on the y-axis. WOMAC score is inverted, higher values represent better health status. All covariates were held constant (mean value for continuous variables; reference level for factors).
