# Supplementary material for: Association of self-efficacy, risk attitudes, and time preferences with health-related quality of life and functioning after total hip or knee replacement – Results of the MobilE-TRA 2 cohort
Source: Health Qual Life Outcomes. 2025 Apr 23;23:44. doi: 10.1186/s12955-025-02374-y (PMC12020169; doi:10.1186/s12955-025-02374-y)
Supplement: Supplementary file 7 — Supplementary Material 7 [file 12955_2025_2374_MOESM7_ESM.docx]

**Supplementary File 7: Table S3: Robustness check of regression results: present orientation instead of future orientation.**

|  | **EQ-5D-5L**  **utility index** | | **EQ-VAS** | | **WOMAC global** | | **WOMAC function** | | **WOMAC pain** | |
| --- | --- | --- | --- | --- | --- | --- | --- | --- | --- | --- |
|  | **β THR** | **β TKR** | **β THR** | **β TKR** | **β THR** | **β TKR** | **β THR** | **β TKR** | **β THR** | **β TKR** |
| Intercept | **0.2903 *** | 0.7096 | **35.1986 *** | **45.6365 *** | **41.4691 **** | **47.4396 **** | 26.999 | **60.136 ***** | **51.217 ***** | **53.8873 ***** |
| Self-efficacy | **0.0682 *** | -0.0066 | 2.8013 | 5.0048 | **6.2659 *** | 0.9208 | **7.8894 **** | -0.1967 | **5.9227 *** | 0.8857 |
| Health-related willingness to take risk | -0.0053 | 0.0127 | -1.4442 | 0.4193 | 0.1025 | 1.4983 | -0.1838 | 1.675 | -0.0847 | 1.5324 |
| Present orientation | 0.0093 | -0.0202 | 2.7958 | -1.6374 | -0.3652 | -1.2584 | 1.2752 | -1.81 | -1.3631 | -1.0504 |
| Male | 0.0335 | -0.0575 | 5.6523 | -3.1087 | 7.3273 | -5.863 | 7.0981 | -6.143 | **9.7903 *** | 0.6991 |
| Age | 0.0032 | -0.0019 | 0.5078 | -0.0002 | 0.3677 | -0.3072 | 0.3037 | -0.372 | 0.54 | -0.2508 |
| Diseases = 1 | **0.1288 *** | -0.0106 | -0.7768 | -1.0648 | -0.4423 | 9.3075 | 5.7053 | 5.7669 | -4.3284 | 4.1511 |
| = 2 | 0.0477 | -0.0246 | -2.9672 | -8.66 | -0.1065 | 9.6859 | 3.6881 | 6.02 | -4.0719 | 6.2521 |
| = 3 | 0.1067 | -0.0006 | -4.2172 | -6.0333 | -2.75 | 11.873 | 3.7884 | 7.1457 | -13.6547 | 6.6844 |
| >= 4 | 0.0312 | -0.1466 | -7.5447 | -8.6662 | -8.9068 | -5.5797 | -4.3647 | -8.5152 | -11.8747 | -6.2709 |
| Years of  Education = 10-11 | 0.0575 | -0.0038 | **18.513 **** | 4.0777 | NA | NA | NA | NA | NA | NA |
| = 12-13 | **-0.1996 *** | -0.013 | -10.0582 | 0.4757 | NA | NA | NA | NA | NA | NA |
| > 13 | -0.0066 | -0.0773 | 5.8798 | -7.132 | NA | NA | NA | NA | NA | NA |
| Outcome at baseline | **-0.6804 ***** | **-0.6768 ***** | **-0.7589 ***** | **-0.7739 ***** | **-0.9152 ***** | **-0.7128 ***** | **-0.8264 ***** | **-0.7322 ***** | **-0.8688 ***** | **-0.7804 ***** |

Each column shows the influence of all included independent variables on the change score of one specific outcome measure (follow-up - baseline). THR: total hip replacement; TKR: total knee replacement; EQ-5D-5L: EuroQol Five-Dimensional Five-Level Questionnaire; EQ-VAS: EuroQol visual analogue scale; WOMAC: Western Ontario and McMaster Universities Osteoarthritis Index; general self-efficacy (1-5): higher scores indicate higher self-efficacy; health-related willingness to take risk (0-10): lower values represent risk aversion; present orientation (1-5): higher values indicate a stronger orientation on the present; male: 0 = female, 1 = male; diseases present at baseline (0-4): number of diseases present at baseline assessment with 4 aggregating counts of 4 diseases and above; outcome at baseline: adjustment for baseline value of the specific outcome.
^a^: In estimations with WOMAC as the outcome, education was not part of the minimal sufficient adjustment set, not included as a covariate, and thus appears as NA in the table.
Significance: *p < 0.05, **p < 0.01, ***p < 0.001; in bold if the result was at least significant at p < 0.05 level.
