# Supplementary material for: Association of self-efficacy, risk attitudes, and time preferences with health-related quality of life and functioning after total hip or knee replacement – Results of the MobilE-TRA 2 cohort
Source: Health Qual Life Outcomes. 2025 Apr 23;23:44. doi: 10.1186/s12955-025-02374-y (PMC12020169; doi:10.1186/s12955-025-02374-y)
Supplement: Supplementary file 8 — Supplementary Material 8 [file 12955_2025_2374_MOESM8_ESM.docx]

**Supplementary File 8: Figure S7: ASA scores plotted against assessment dates.**


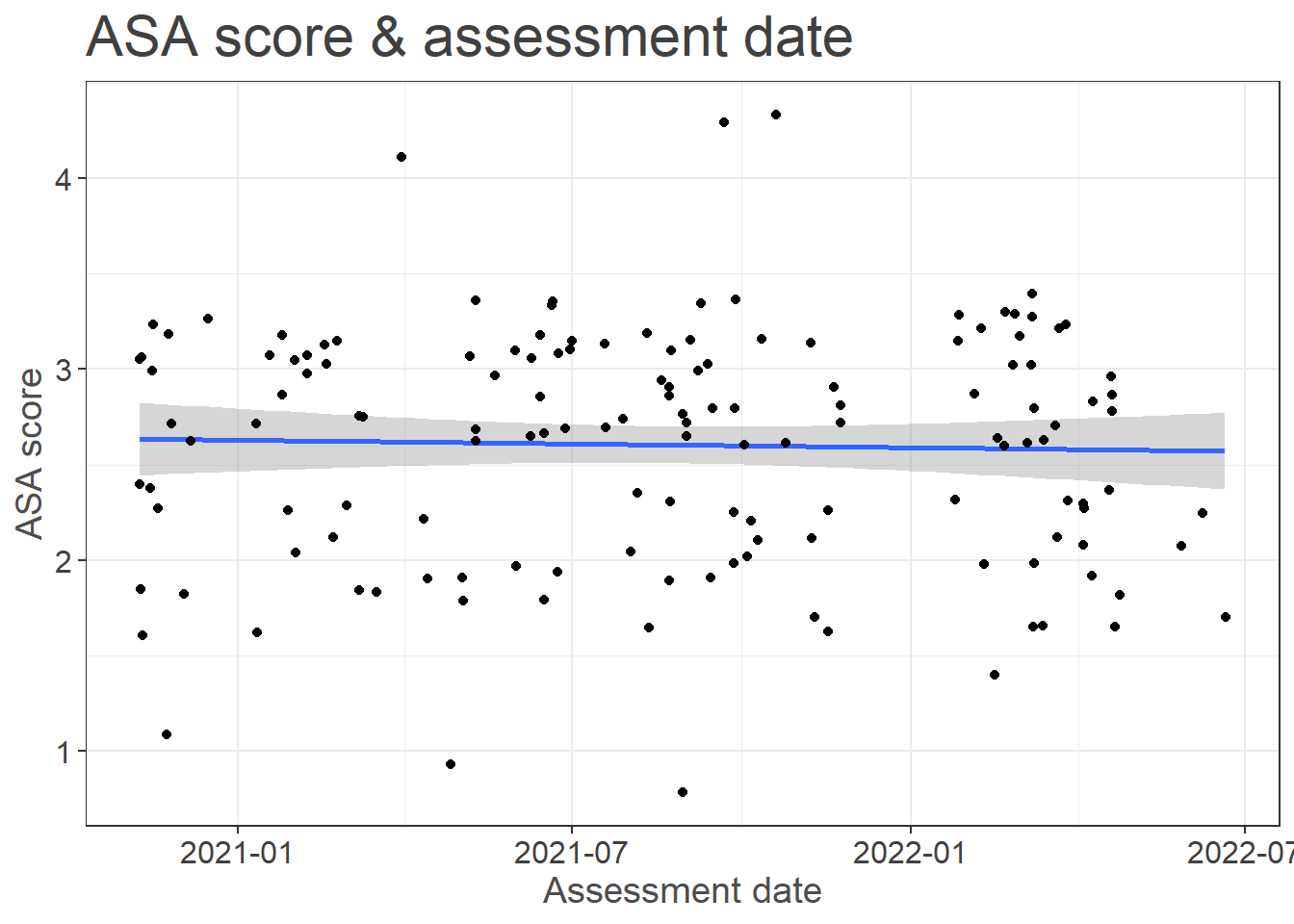


Time trend analysis of patient status to determine if the SARS-CoV-2 pandemic induced a selection bias. ASA score (1-5): American Society of Anesthesiologists score assessing the physical status of a patient before surgery with values representing (1) healthy, (2) mild systemic disease, (3) severe systemic disease, (4) life-threatening severe systemic disease, (5) moribund patient respectively. The assessment date is positioned on the x-axis, and the American Association of Anesthesiologists (ASA) score is on the y-axis. The solid line depicts the regressed association computed in a linear model. Individual assessments are depicted by dots. Plotting of dots occurred with jitter to avoid overlap. The light grey bars highlight two recruitment periods with strong surgery reductions due to SARS-CoV-2 pandemic measures.
